# Tree-based Node Aggregation in Sparse Graphical Models

**Published:** 2022-09

**Authors:** Ines Wilms, Jacob Bien

**Affiliations:** Department of Quantitative Economics, Maastricht University, Maastricht, The Netherlands; Department of Data Sciences and Operations, Marshall School of Business, University of Southern California, California, USA

**Keywords:** aggregation, graphical model, high-dimensionality, regularization, sparsity

## Abstract

High-dimensional graphical models are often estimated using regularization that is aimed at reducing the number of edges in a network. In this work, we show how even simpler networks can be produced by aggregating the nodes of the graphical model. We develop a new convex regularized method, called the *tree-aggregated graphical lasso* or tag-lasso, that estimates graphical models that are both edge-sparse and node-aggregated. The aggregation is performed in a data-driven fashion by leveraging side information in the form of a tree that encodes node similarity and facilitates the interpretation of the resulting aggregated nodes. We provide an efficient implementation of the tag-lasso by using the locally adaptive alternating direction method of multipliers and illustrate our proposal’s practical advantages in simulation and in applications in finance and biology.

## 1. Introduction

Graphical models are greatly useful for understanding the relationships among large numbers of variables. Yet, estimating graphical models with many more parameters than observations is challenging, which has led to an active area of research on high-dimensional inverse covariance estimation. Numerous methods attempt to curb the curse of dimensionality through regularized estimation procedures (e.g., Meinshausen and Bühlmann, 2006; Yuan and Lin, 2007; Banerjee et al., 2008; Friedman et al., 2008; Rothman et al., 2008; Peng et al., 2009; Yuan, 2010; Cai et al., 2011, 2016). Such methods aim for sparsity in the inverse covariance matrix, which corresponds to graphical models with only a small number of edges. A common method for estimating sparse graphical models is the graphical lasso (glasso) (Yuan and Lin, 2007; Banerjee et al., 2008; Rothman et al., 2008; Friedman et al., 2008), which adds an $\ell_{1}$-penalty to the negative log-likelihood of a sample of multivariate normal random variables. While this and many other methods focus on the *edges* for dimension reduction, far fewer contributions (e.g., Tan et al., 2015; Eisenach et al., 2020; Pircalabelu and Claeskens, 2020) focus on the *nodes* as a guiding principle for dimension reduction.

Nonetheless, node dimension reduction is becoming increasingly relevant in many areas where data are being measured at finer levels of granularity. For instance, in biology, modern high-throughput sequencing technologies provide low-cost microbiome data at high resolution; in neuroscience, brain activity in hundreds of regions of interest can be measured; in finance, data at the individual company level at short time scales are routinely analyzed; and in marketing, joint purchasing data on every stock-keeping-unit (product) are recorded. The fine-grained nature of these data brings new challenges. The sheer number of fine-grained, often noisy, variables makes it difficult to detect dependencies. Moreover, there can be a mismatch between the resolution of the measurement and the resolution at which natural meaningful interpretations can be made. The purpose of an analysis may be to draw conclusions about entities at a coarser level of resolution than happened to be measured. Because of this mismatch, practitioners are sometimes forced to devise ad hoc post-processing steps involving, for example, coloring the nodes based on some classification of them into groups in an attempt to make the structure of an estimated graphical model more interpretable and the domain-specific takeaways more apparent (e.g., Millington and Niranjan, 2019).

Our solution to this problem is to incorporate the side information about the relationship between nodes directly into the estimation procedure. In our framework, this side information is encoded as a tree whose leaves correspond to the measured variables. Such tree structures are readily available in many domains (e.g., taxonomies in biology and hierarchical classifications of jobs, companies, and products in business) and is well-suited to expressing the multi-resolution structure that is present in many problems. We propose a new convex regularization procedure, called *tag-lasso*, which stands for *tree-aggregated-graphical*-*lasso*. This procedure combines node (or variable) aggregation with edge-sparsity. The tree-based aggregation serves to both amplify the signal of similar, low-level variables and render a graphical model involving nodes at an appropriate level of scale to be relevant and interpretable. The edge-sparsity encourages the graphical model involving the aggregated nodes to have a sparse network structure.

**Figure 1:**
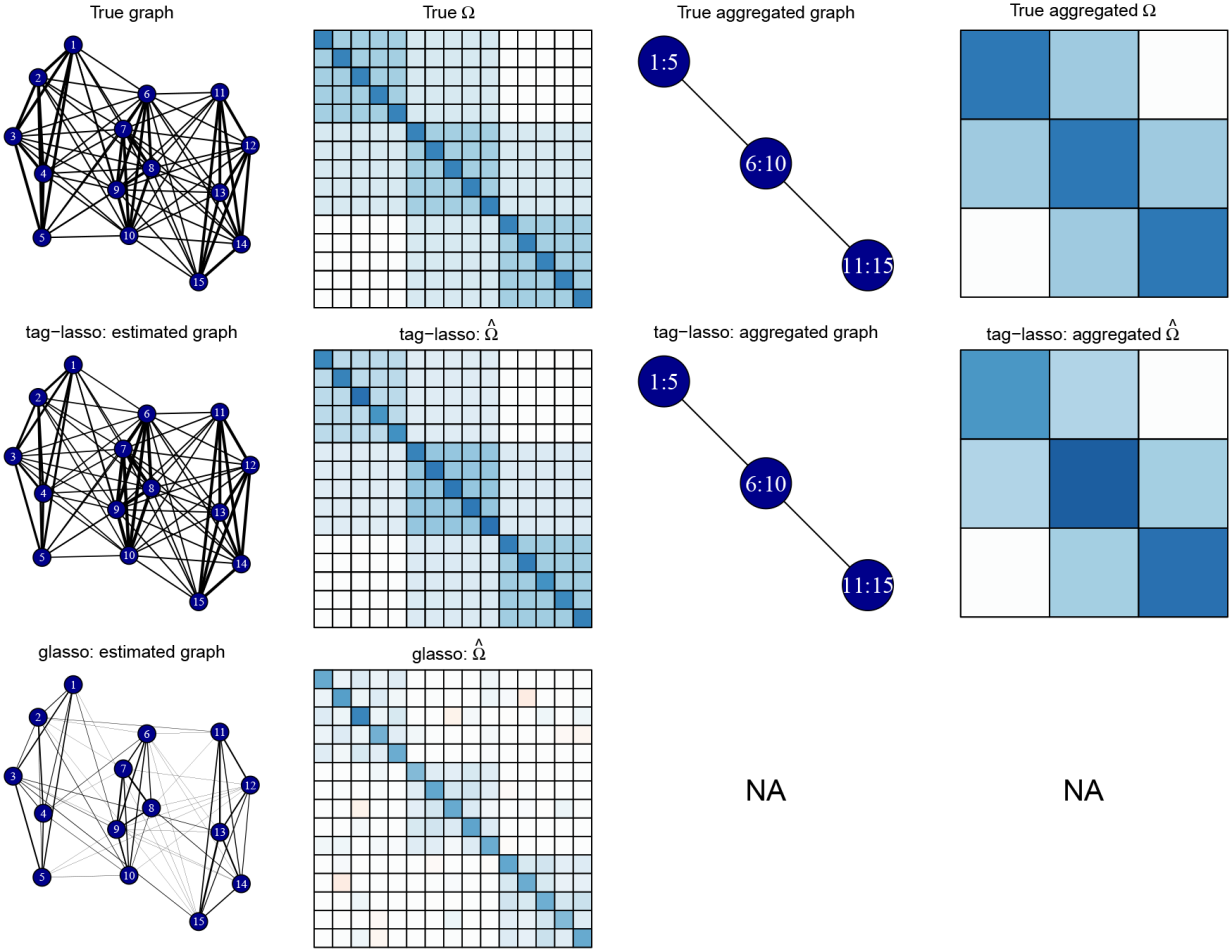
Top: True graph and precision matrix Ω with corresponding aggregated graph and precision matrix. Middle: Estimation output of the tag-lasso. Bottom: Estimation output of the glasso.

Our procedure is based on a tree-based parameterization strategy that translates the node aggregation problem into a sparse modeling problem, following an approach previously introduced in the regression setting (Yan and Bien, 2021). In Figure 1 (to be discussed more thoroughly in Section 4), we see that tag-lasso is able to recover the aggregated, sparse graph structure. By doing so, it yields a more accurate estimate of the true graph, and its output is easier to interpret than the full, noisy graph obtained by the glasso.

The rest of the paper is organized as follows. Section 2 introduces the tree-based parameterization structure for nodewise aggregation in graphical models. Section 3 introduces the tag-lasso estimator, formulated as a solution to a convex optimization problem, for which we derive an efficient algorithm. Section 4 presents the results of a simulation study. Section 5 illustrates the practical advantages of the tag-lasso on financial and microbiome data sets. Section 6 concludes.

## 2. Node Aggregation in Penalized Graphical Models

Let S be the empirical covariance matrix based on n multivariate normal observations of dimension p, with mean vector μ and covariance matrix Σ. The target of estimation is the precision matrix $\Omega =\Sigma^{-1}$, whose sparsity pattern provides the graph structure of the Gaussian graphical model, since $\Omega_{jk}=0$ is equivalent to variables j and k being conditionally independent given all other variables. To estimate the precision matrix, it is common to use a convex penalization method of the form

(1) $$\hat{\Omega }=\underset{\Omega }{\text{argmin}}\left\{-\text{logdet}\left(\Omega \right)+\text{tr}\left(S\Omega \right)+\lambda 𝒫\left(\Omega \right)\text{ s}.\text{t}.\;\Omega =\Omega^{⊤},\Omega ≻0\right\},$$

where tr(⋅) denotes the trace, ·≻0 denotes a positive definite matrix, 𝒫(⋅) is a convex penalty function, and λ>0 is a tuning parameter controlling the degree of penalization. Choosing the $\ell_{1}$-norm

(2) $$𝒫\left(\Omega \right)={\left\|\Omega^{-\text{diag}}\right\|}_{1},$$

where $\Omega^{-\text{diag }}$ contains the unique off-diagonal elements, yields the *graphical lasso* (glasso) (Friedman et al., 2008; Yuan and Lin, 2007; Banerjee et al., 2008; Rothman et al., 2008). It encourages $\hat{\Omega }$ to be sparse, corresponding to a graphical model with few edges.

However, when Ω is not sparse, demanding sparsity in $\hat{\Omega }$ may not be helpful, as we will show in Section 2.1. Such settings can arise when data are measured and analyzed at ever higher resolutions (a growing trend in many areas, see e.g. Callahan et al. 2017). A tree is a natural way to represent the different scales of data resolution, and we introduce a new choice for 𝒫 that uses this tree to guide node aggregation, thereby allowing for a data adaptive choice of data scale for capturing dependencies. Such tree-based structures are available in many domains. For instance, companies can be aggregated according to hierarchical industry classification codes; products can be aggregated from brands towards product categories; brain voxels can be aggregated according to brain regions; microbiome data can be aggregated according to taxonomy. The resulting penalty function then encourages a more general and yet still highly interpretable structure for $\hat{\Omega }$. In the following subsection, we use a toy example to illustrate the power of such an approach.

### 2.1. Node Aggregation

Consider a toy example with p variables

$$X_{1}=\overset{p}{\underset{j=3}{\sum }}X_{j}+\varepsilon_{1}$$

$$X_{2}=\overset{p}{\underset{j=3}{\sum }}X_{j}+\varepsilon_{2}$$

$$X_{j}=\varepsilon_{j},\text{ for }3\leq j\leq p,$$

where $\varepsilon_{1},\ldots ,\varepsilon_{p}$ are independent standard normal random variables. By construction, it is clear that there is a very simple relationship between the variables: The first two variables both depend on the sum of the other p−2 variables. However, a standard graphical model on the p variables does not naturally express this simplicity. The first row of Table 1 shows the covariance and precision matrices for the full set of variables $X_{1},\ldots ,X_{p}$. The graph in the last column then visually represents the same information as the precision matrix. While this graph does convey the message that variables 1 and 2 are conditionally independent, it is extremely dense with $O\left(p^{2}\right)$ edges. As such, the precise structure among the remaining variables is hard to infer from the graph, an issue that only becomes worse when the number of variables p increases. Imagine if instead we could form a graphical model with only three variables: $X_{1},X_{2},\tilde{X}$, where the last variable $\tilde{X}=\sum_{j=3}^{p}X_{j}$ aggregates all but the first two variables. The bottom row of Table 1 results in a graphical model that matches the simplicity of the situation. The graph with aggregated nodes maintains its simplicity even when p increases.

The lack of sparsity in the p-node graphical model means that the graphical lasso will not do well; its estimation accuracy will suffer unless the sample size is extremely large. Nonetheless, a method that could perform node aggregation would be able to yield a highlyinterpretable aggregated sparse graphical model since $X_{1}$ and $X_{2}$ are conditionally independent given the aggregated variable $\tilde{X}$.

It is useful to map from the small aggregated graphical model to the original p-node graphical model. One does so by writing the precision matrix in “G-block” format (Bunea et al., 2020, although they introduce this terminology in the context of the covariance matrix, not its inverse) for a given partition $G=\left\{G_{1},\ldots ,G_{K}\right\}$ of the nodes {1,…,p} and corresponding p×K membership matrix M, with entries $M_{jk}=1$ if $j\in G_{k}$, and $M_{jk}=0$ otherwise. In particular, there exists a K×K symmetric matrix C and a p×p diagonal matrix D such that the precision matrix can be written as $\Omega =MCM^{⊤}+D$. The block-structure of Ω is captured by the first part of the decomposition, the aggregated K×K precision matrix on the set of aggregated nodes can then be written as $\Omega_{\text{agg}}=C+D_{\text{agg}}$, where $D_{\text{agg}}={\left(M^{⊤}D^{-1}M\right)}^{-1}$ is diagonal. In the above example, K=3, $G_{1}=\left\{1\right\}$, $G_{2}=\left\{2\right\}$, $G_{3}=\left\{3,\ldots ,p\right\}$ and $MCM^{⊤}$ has only three distinct rows/columns since the aggregated variables j=3,…,p share all their entries. In the presence of node aggregation and edge sparsity, the graphical model corresponding to the aggregated precision matrix is far more parsimonious than the graphical model on the full precision matrix (see Table 1).

As motivated by this example, our main goal is to estimate the precision matrix in such a way that we can navigate from a p-dimensional problem to a K-dimensional problem whose corresponding graphical model provides a simple description of the conditional dependency structure among K aggregates of the original variables. In the following proposition, we show that this can be accomplished by looking for a precision matrix that has a G-block structure. The proof of the proposition is included in Appendix A.

**Proposition 1** *Suppose* $X\sim {\text{N}}_{\text{p}}\left(0,\Omega^{-1}\right)$ *with $\Omega =MCM^{⊤}+D$*, *where $M\in {\left\{0,1\right\}}^{p\times K}$ is the membership matrix*, D≻0*, and let $\tilde{X}=M^{⊤}X\in {\mathbb{R}}^{\text{K}}$ be the vector of aggregated variables. Then $\tilde{X}$ has precision matrix $\Omega_{\text{agg}}=C+D_{\text{agg}}$, where $D_{\text{agg}}$ is a diagonal matrix, and therefore* ${\text{c}}_{\text{ij}}=0$ i*s equivalent to the aggregates ${\tilde{X}}_{\text{i}}$ and ${\tilde{X}}_{\text{j}}$ being conditionally independent given all other aggregated variables*.

The same matrix C thus enters the formula for Ω as well as $\Omega_{\text{agg}}$, thereby ensuring that both reflect the same conditional independence structure. While Proposition 1 thereby gives us the desired interpretation in the graphical model with K aggregated nodes, in practice, the partition G, its size K, and corresponding membership matrix M are, however, unknown. Rather than considering arbitrary partitions of the variables, we constrain ourselves specifically to partitions guided by a known tree. In so doing, we allow ourselves to exploit side information and help ensure that the aggregated nodes will be easily interpretable. To this end, we introduce a tree-based parameterization strategy that allows us to embed the node dimension reduction into a convex optimization framework.

### 2.2. Tree-Based Parameterization

**Figure 2:**
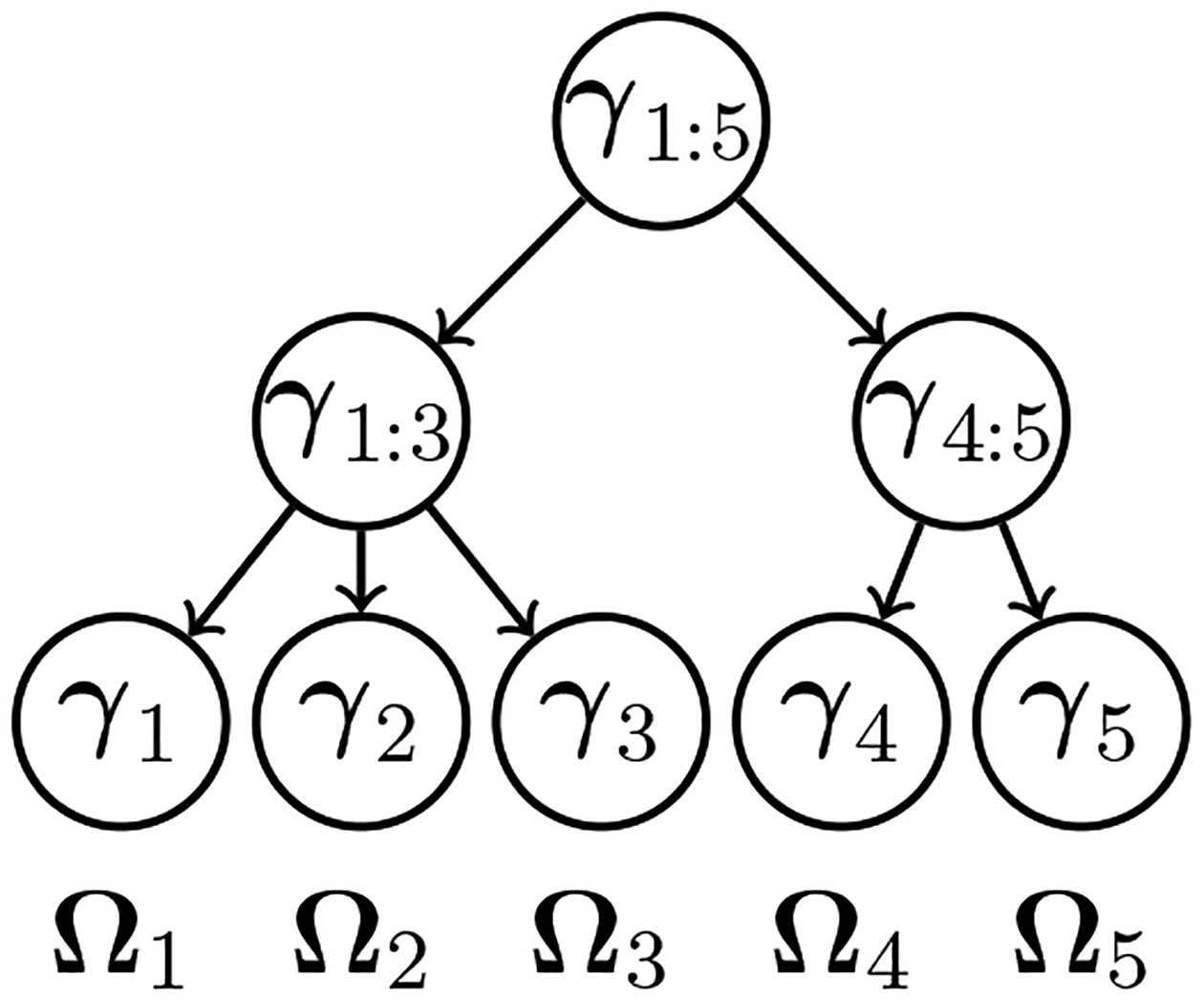
An example of a tree 𝒯 encoding similarity among p=5 variables.

Our aggregation procedure assumes that we have, as side information, a tree that represents the closeness (or similarity) of variables. We introduce here a matrix-valued extension of the tree-based parameterization developed in Yan and Bien (2021) for the regression setting. We consider a tree 𝒯 with p leaves $\Omega_{1},\ldots ,\Omega_{p}$ where $\Omega_{j}$ denotes column 1≤j≤p of Ω. We restrict ourselves to partitions that can be expressed as a collection of branches of 𝒯. Newly aggregated nodes are then formed by summing variables within branches. To this end, we assign a p-dimensional parameter vector $\gamma_{u}$ to each node u in the tree 𝒯 (see Figure 2 for an example). Writing the set of nodes in the path from the root to the $j^{\text{th }}$ leaf (variable) as ancestor (j)∪{j}, we express each column/row in the precision matrix as

(3) $$\Omega_{j}=\underset{u\in \text{ancestor}\left(j\right)\cup \left\{j\right\}}{\sum }\gamma_{u}+d_{j}e_{j},$$

where we sum over all the $\gamma_{u}$ ‘s along this path, and $e_{j}$ denotes the p-dimensional vector with all zeros except for its $j^{\text{th }}$ element that is equal to one. In the remainder, we will make extensive use of the more compact notation Ω=AΓ+D, where $A\in {\left\{0,1\right\}}^{p\times \left|𝒯\right|}$ is a binary matrix with $A_{jk}=1\left\{u_{k}\in \text{ancestor}\left(j\right)\cup \left\{j\right\}\right\}=1\left\{j\in \text{descendant}\left(u_{k}\right)\cup \left\{u_{k}\right\}\right\}$ with 1{⋅} denoting the indicator function, Γ is a |𝒯|×p parameter matrix collecting the $\gamma_{u}$ ‘s in its rows with |𝒯| denoting the cardinality of the tree and D is a diagonal parameter matrix with elements $d_{1},\ldots ,d_{p}$.

**Figure 3:**
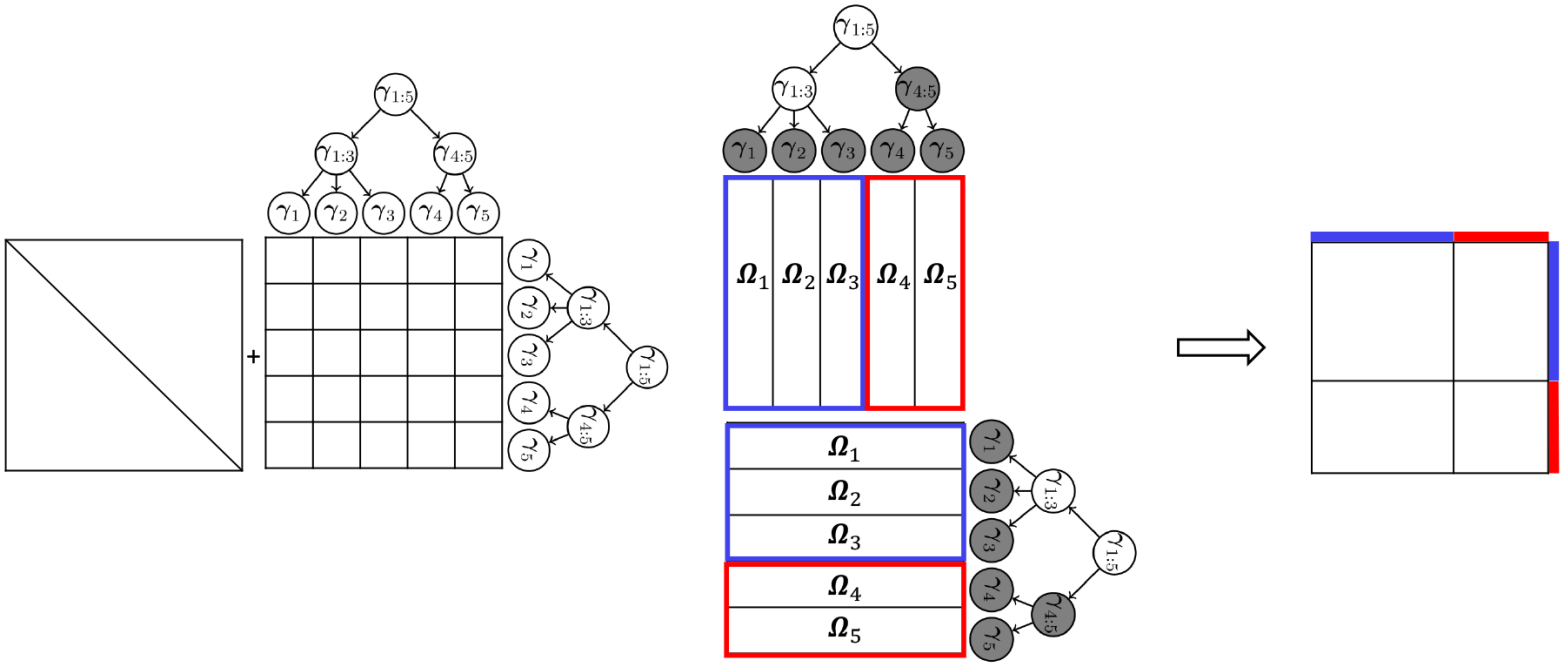
Left: An example of a 5×5-dimensional Ω and a tree 𝒯 that relates the corresponding p=5 variables. We have $\Omega_{i}=\gamma_{i}+\gamma_{1:3}+\gamma_{1:5}$ for i=1,2,3 and $\Omega_{j}=\gamma_{j}+\gamma_{4:5}+\gamma_{1:5}$ for j=4,5, by equation (3), ignoring the diagonal elements. Middle: By zeroing out the $\gamma_{i}$ ‘s in the gray nodes, we aggregate the rows/columns of Ω into two groups indicated by the two colors: $\Omega_{1}=\Omega_{2}=\Omega_{3}=\gamma_{1:3}+\gamma_{1:5}$ (blue) and $\Omega_{4}=\Omega_{5}=\gamma_{1:5}$ (red). Right: The precision matrix Ω thus has a block-structure.

By zeroing out γu's, certain nodes will be aggregated, as can be seen from the illustrative example in Figure 3. More precisely, let $𝒱=\left\{u:\gamma_{u}\neq 0\right\}$ denote the set of non-zero rows in Γ and let $A_{𝒱}$ be the sub-matrix of A where only the columns corresponding to the non-zero rows in Γ are kept. The number of blocks K in the aggregated network is then given by the number of unique rows in $A_{𝒱}$. The membership matrix M (Section 2.1), and hence the set of aggregated nodes, can then be derived from the variables (rows) in the matrix $A_{𝒱}$ that share all their row-entries. In the next section, we introduce the tag-lasso, which is based on this parameterization.

We prefer to perform aggregation according to tree-based structures, as opposed to more general directed acyclic graphs, because of the interpretability trees naturally offer. For trees, each node has only one parent and, hence, there exists a single path from the leaves (original variables) towards the root node (complete aggregation). In such cases, the intermediate levels in the tree provide a direct, natural labelling of the corresponding aggregated variables. We discuss the option to perform aggregation guided by more general graph-based structures in Section 6.

## 3. Tree Aggregated Graphical lasso

To achieve dimension reduction via node aggregation and edge sparsity simultaneously, we extend optimization problem (1) by incorporating the parameterization introduced above. Our estimator, called the *tag-lasso*, is defined as

(4) $$\left(\hat{\Omega },\hat{\Gamma },\hat{D}\right)=\underset{\Omega ,\Gamma ,D}{\text{argmin}}\{-\text{logdet}\left(\Omega \right)+\text{tr}\left(S\Omega \right)+\lambda_{1}{\left\|\Gamma_{-r}\right\|}_{2,1}+\lambda_{2}{\left\|\Omega^{-\text{diag}}\right\|}_{1}\text{ s}.\text{t}.\;\Omega =\Omega^{⊤},\Omega ≻0,\gamma_{r}=\gamma 1_{p},\Omega =A\Gamma +D,D\text{ diag},D_{jj}\geq 0\text{ for }j=1,\ldots ,p\},$$

with ${\left\|\Gamma_{-r}\right\|}_{2,1}=\sum_{u\in {𝒯}_{-r}}{\left\|\gamma_{u}\right\|}_{2}$ and ${𝒯}_{-r}$ being the set of all nodes in 𝒯 other than the root. This norm induces row-wise sparsity on all non-root rows of Γ. This row-wise sparsity, in turn, induces node aggregation as explained in Section 2.2. The root is excluded from this penalty term so that in the extreme case of a large $\lambda_{1}$ one gets complete aggregation but not necessarily sparsity (in this extreme, all off-diagonal elements of $\hat{\Omega }$ are equal to the scalar γ that appears in the equality constraint involving $\gamma_{r}$). While $\lambda_{1}$ controls the degree of node aggregation, $\lambda_{2}$ controls the degree of edge sparsity. When $\lambda_{1}=0$, the optimization problem in (4) reduces to the glasso.

The tag-lasso estimator imposes a block structure on the rows of $\hat{\Omega }-\hat{D}$ via the constraint Ω=AΓ+D. From the sparsity pattern of $\hat{\Gamma }$, we obtain $\hat{𝒱}=\left\{u\in 𝒯:{\hat{\gamma }}_{u}\neq 0\right\}$, the set of non-zero rows in $\hat{\Gamma }$, such that we can write $\hat{\Omega }-\hat{D}=A\hat{\Gamma }=A_{\hat{𝒱}}{\hat{\Gamma }}_{\hat{𝒱}}$. Algorithm 1 in Appendix A details how the membership matrix $\hat{M}$ can be obtained from the original binary matrix A and the set $\hat{𝒱}$. Due to the enforced symmetry on the precision matrix, the block structure imposed on the rows of $\hat{\Omega }-\hat{D}$ holds likewise for its columns. The next proposition then shows how the solution provided by the tag-lasso can be re-written in G-block format. The proof of the proposition is included in Appendix A.

**Proposition 2** *Given a solution* $\left(\hat{\Omega },\hat{\Gamma },\hat{D}\right)$ *to the tag-lasso problem, there exists* a p×K *partition matrix* $\hat{M}$ *and a symmetric* K×K *matrix $\hat{C}$ such that $\hat{\Omega }=\hat{M}\hat{C}{\hat{M}}^{⊤}+\hat{D}$*.

The G-block structure imposed by the tag-lasso (in case K<p) implies that the tag-lasso is especially useful to apply when one believes that dimension reduction can be leveraged in terms of node-aggregation in addition to edge-sparsity.

Finally, note that optimization problem (4) fits into the general formulation of penalized graphical models given in (1) since it can be equivalently expressed as

$$\hat{\Omega }=\underset{\Omega }{\text{argmin}}\left\{-\text{logdet}\left(\Omega \right)+\text{tr}\left(S\Omega \right)+\lambda_{1}{𝒫}_{\text{aggregate }}\left(\Omega \right)+\lambda_{2}{𝒫}_{\text{sparse }}\left(\Omega \right)\text{ s}.\text{t}.\;\Omega =\Omega^{⊤},\Omega ≻0\right\},$$

where

$${𝒫}_{\text{aggregate }}\left(\Omega \right)=\underset{\Gamma ,D}{\text{min}}\left\{{\left\|\Gamma_{-r}\right\|}_{2,1}\text{s}.\text{t}.\;\gamma_{r}=\gamma 1_{p},\Omega =A\Gamma +D,D\text{ diag},D_{jj}\geq 0\text{ for }j=1,\ldots ,p\right\}$$

and ${𝒫}_{\text{sparse }}\left(\Omega \right)$ is the $\ell_{1}$-norm defined in (2).

The proposed tag-lasso estimator thus determines the node aggregation and edge sparsity while also producing an estimate of the precision matrix by solving one convex optimization problem. One might wonder how such a “one-stage” procedure compares to a two-stage procedure where first the level of node aggregation is determined, and secondly, the glasso is applied using the aggregated nodes determined in the first stage. In Appendix B we detail this two-stage procedure and compare its performance to the tag-lasso estimator through a simulation study. Across various simulation designs, we find that the proposed (one-stage) tag-lasso estimator provides important improvements in terms of estimation accuracy over such a two-stage benchmark as the latter struggles to retrieve the correct aggregation level.

### 3.1. Locally Adaptive Alternating Direction Method of Multipliers

We develop an *alternating direction method of multipliers* (ADMM) algorithm (Boyd et al., 2011), specifically tailored to solving (4). Our ADMM algorithm is based on solving this equivalent formulation of (4) :

(5) $$\underset{\begin{array}{l} \Omega^{\left(1\right)},\Omega^{\left(2\right)},\Omega^{\left(3\right)}\\ \Gamma^{\left(1\right)},\Gamma^{\left(2\right)},\Omega ,\Gamma ,D \end{array}}{\text{min}}\{-\text{logdet}\left(\Omega^{\left(1\right)}\right)+\text{tr}\left(S\Omega^{\left(1\right)}\right)+\lambda_{1}{\left\|\Gamma_{-r}^{\left(1\right)}\right\|}_{2,1}+\lambda_{2}{\left\|\Omega^{-\text{diag}\left(3\right)}\right\|}_{1}\text{ s}.\text{t}.\;\Omega^{\left(1\right)}=\Omega^{{\left(1\right)}^{⊤}},\Omega^{\left(1\right)}≻0,\gamma_{r}^{\left(1\right)}=\gamma^{\left(1\right)}1_{p},\Omega^{\left(2\right)}=A\Gamma^{\left(2\right)}+D,D\text{ diag},D_{jj}\geq 0,j=1,\ldots ,p,\Omega =\Omega^{\left(1\right)}=\Omega^{\left(2\right)}=\Omega^{\left(3\right)}\text{ and }\Gamma =\Gamma^{\left(1\right)}=\Gamma^{\left(2\right)}\}.$$

Additional copies of Ω and Γ are introduced to efficiently decouple the optimization problem.

Furthermore, we use an extension called *locally adaptive-ADMM* (LA-ADMM, Xu et al., 2017) with adaptive penalization to improve performance. The full details of the algorithm are provided in Appendix C. The computational complexity of the algorithm in terms of the number of variables p and the size of the tree |𝒯| is $𝒪\left(p|𝒯{|}^{2}\right)\times \left(\text{number of iterations}+1\right)$ since each initialization step as well as each per iteration update is at most $𝒪\left(p|𝒯{|}^{2}\right)$. Bounds on the number of iterations for ADMM and LA-ADMM can be found in Xu et al. (2017).

### 3.2. Selection of the Tuning Parameters

To select the tuning parameters $\lambda_{1}$ and $\lambda_{2}$, we form a 10 × 10 grid of $\left(\lambda_{1},\lambda_{2}\right)$ values and find the pair that minimizes a 5-fold cross-validated likelihood-based score,

(6) $$\frac{1}{5}\overset{5}{\underset{k=1}{\sum }}\left\{-\text{logdet}\left({\hat{\Omega }}_{-{ℱ}_{k}}\right)+\text{tr}\left(S_{{ℱ}_{k}}{\hat{\Omega }}_{-{ℱ}_{k}}\right)\right\},$$

where ${\hat{\Omega }}_{-{ℱ}_{k}}$ is an estimate of the precision matrix trained while withholding the samples in the $k^{\text{th }}$ fold and $S_{{ℱ}_{k}}$ is the sample covariance matrix computed on the $k^{\text{th }}$ fold. In particular, we take ${\hat{\Omega }}_{-{ℱ}_{k}}$ to be a re-fitted version of our estimator (e.g., Belloni and Chernozhukov, 2013). After fitting the tag-lasso, recall that we obtain $\hat{𝒱}=\left\{u\in 𝒯:{\hat{\gamma }}_{u}\neq 0\right\}$, the set of non-zero rows in $\hat{\Gamma }$, which suggests a particular node aggregation; and $\hat{ℰ}=\left\{\left(i,j\right):{\hat{\Omega }}_{ij}\neq 0\right\}$, the set of non-zero elements in $\hat{\Omega }$, which suggests a particular edge sparsity structure. We then re-estimate Ω by maximizing the likelihood subject to these aggregation and sparsity constraints:

(7) $$\begin{array}{ll} \underset{\Omega ,\Gamma_{\hat{𝒱}},D}{\text{min}} & -\text{logdet}\left(\Omega \right)+\text{tr}\left(S\Omega \right)\\ \text{ subject to } & \Omega =\Omega^{⊤},\Omega ≻0,\\ \; & \gamma_{\hat{𝒱},r}=\gamma 1_{p},\\ \; & \Omega =A_{\hat{𝒱}}\Gamma_{\hat{𝒱}}+D,D\text{ diag}.,\;D_{jj}\geq 0\text{ for }j=1,\ldots ,p\\ \; & \Omega_{ij}=0,\text{ for }\left(i,j\right)\notin \hat{ℰ}. \end{array}$$

We solve this with an LA-ADMM algorithm similar to what is described in Section 3.1 and Appendix C

### 3.3. Connections to Related Work

Combined forms of dimension reduction in graphical models can be found in, amongst others, Chandrasekaran et al. (2012); Tan et al. (2015); Eisenach et al. (2020); Brownlees et al. (2020); Pircalabelu and Claeskens (2020).

Chandrasekaran et al. (2012) consider a blend of principal component analysis with graphical modeling by combining sparsity with a low-rank structure. Tan et al. (2015) and Eisenach et al. (2020) both propose two-step procedures that first cluster variables in an initial dimension reduction step and subsequently estimate a cluster-based graphical model. Brownlees et al. (2020) introduce partial correlation network models with community structures but rely on the sample covariance matrix of the observations to perform spectral clustering. Our procedure differs from these works by introducing a single convex optimization problem that simultaneously induces aggregation and edge sparsity for the precision matrix.

Our work is most closely related to Pircalabelu and Claeskens (2020) who estimate a penalized graphical model and simultaneously classify nodes into communities. However, Pircalabelu and Claeskens (2020) do not use tree-based node-aggregation. Our approach, in contrast, considers the tree 𝒯 as an important part of the problem to help determine the extent of node aggregation, and as a consequence the number of aggregated nodes (i.e. clusters, communities or blocks) K, in a data-driven way through guidance of the tree-based structure on the nodes.

## 4. Simulations

We investigate the advantages of jointly exploiting node aggregation and edge sparsity in graphical models. To this end, we compare the performance of the tag-lasso to two benchmarks:

- *oracle*: The aggregated, sparse graphical model in (7) is estimated subject to the true aggregation and sparsity constraints. The oracle is only available for simulated data and serves as a “best case” benchmark.
- *glasso*: This does not perform any aggregation (corresponding to the tag-lasso with $\lambda_{1}=0$). A sparse graph on the full set of variables is estimated. The glasso is computed using the same LA-ADMM algorithm as detailed in Appendix C. The tuning parameter is selected from a 10-dimensional grid as the value that minimizes the 5-fold cross-validation likelihood-based score in equation (6) with ${\hat{\Omega }}_{-{ℱ}_{k}}$ taken to be the glasso estimate.

All simulations were performed using the simulator package (Bien, 2016) in R (R Core Team, 2017). We evaluate the estimators in terms of three performance metrics: estimation accuracy, aggregation performance, and sparsity recovery. We evaluate* estimation accuracy* by averaging over many simulation runs the Kullback-Leibler (KL) distance

$$\text{KL}=-\text{logdet}\left(\Sigma \hat{\Omega }\right)+\text{tr}\left(\Sigma \hat{\Omega }\right)-p,$$

where $\Sigma =\Omega^{-1}$ is the true covariance matrix. Note that the KL distance is zero if the estimated precision matrix equals the true precision matrix.

To evaluate *aggregation performance*, we use two measures: the Rand index (Rand, 1971) and the adjusted Rand index (Hubert and Arabie, 1985). Both indices measure the degree of similarity between the true partition on the set of nodes 1,…,p and the estimated partition. The Rand index ranges from zero to one, where one means that both partitions are identical. The adjusted Rand index performs a re-scaling to account for the fact that random chance will cause some variables to occupy the same group.

Finally, to evaluate *sparsity recovery*, we use the false positive and false negative rates

$$\text{FPR}=\frac{\#\left\{\left(i,j\right):{\hat{\Omega }}_{ij}\neq 0\text{ and }\Omega_{ij}=0\right\}}{\#\left\{\left(i,j\right):\Omega_{ij}=0\right\}}\text{ and FNR}=\frac{\#\left\{\left(i,j\right):{\hat{\Omega }}_{ij}=0\text{ and }\Omega_{j}\neq 0\right\}}{\#\left\{\left(i,j\right):\Omega_{ij}\neq 0\right\}}\text{. }$$

The FPR reports the fraction of truly zero components of the precision matrix that are estimated as nonzero. The FNR gives the fraction of truly nonzero components of the precision matrix that are estimated as zero.

### 4.1. Simulation Designs

**Figure 4:**
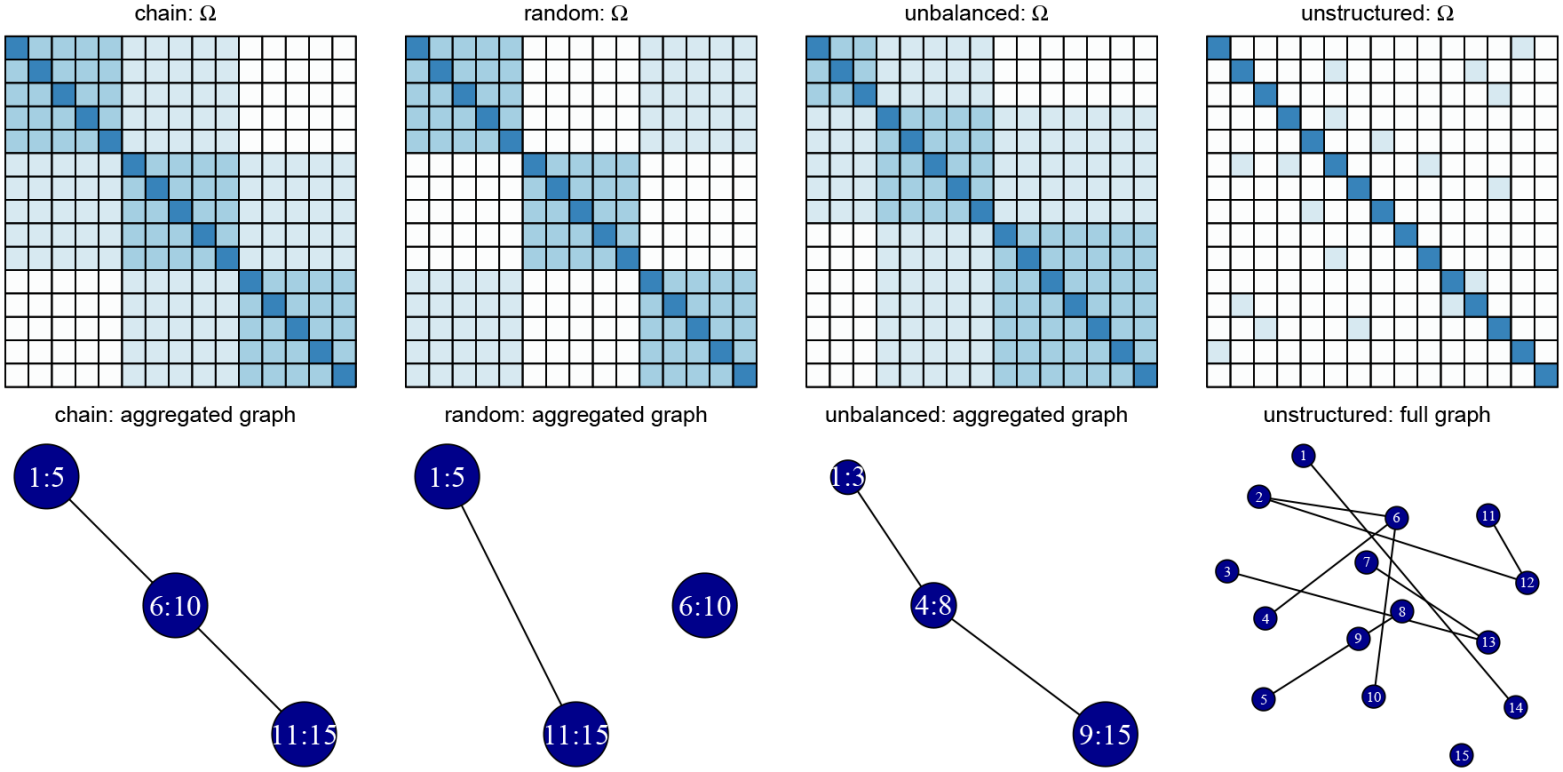
Four aggregation designs: chain, random, unbalanced and unstructured graphs with corresponding precision matrix (top) and graph on the set of aggregated nodes (bottom).

Data are drawn from a multivariate normal distribution with mean zero and covariance matrix $\Sigma =\Omega^{-1}$. We take p=15 variables and investigate the effect of increasing the number of variables in Section 4.3. We consider four different simulation designs, shown in Figure 4, each having a different combination of aggregation and sparsity structures for the precision matrix Ω.

Aggregation is present in the first three structures. The precision matrix has a G-block structure with K=3 blocks. In Section 4.4, we investigate the effect of varying the number of blocks. In the *chain* graph, adjacent aggregated groups are connected through an edge. This structure corresponds to the motivating example of Section 1. In the *random* graph, one non-zero edge in the aggregated network is chosen at random. In the * unbalanced* graph, the clusters are of unequal size. In the *unstructured* graph, no aggregation is present.

Across all designs, we take the diagonal elements of Ω to be 1, the elements within a block of aggregated variables to be 0.5, and the non-zero elements across blocks to be 0.25. We generate 100 different data sets for every simulation design and use a sample size of n=120. The number of parameters (p+p(p−1)/2=120) equals the sample size.

**Figure 5:**
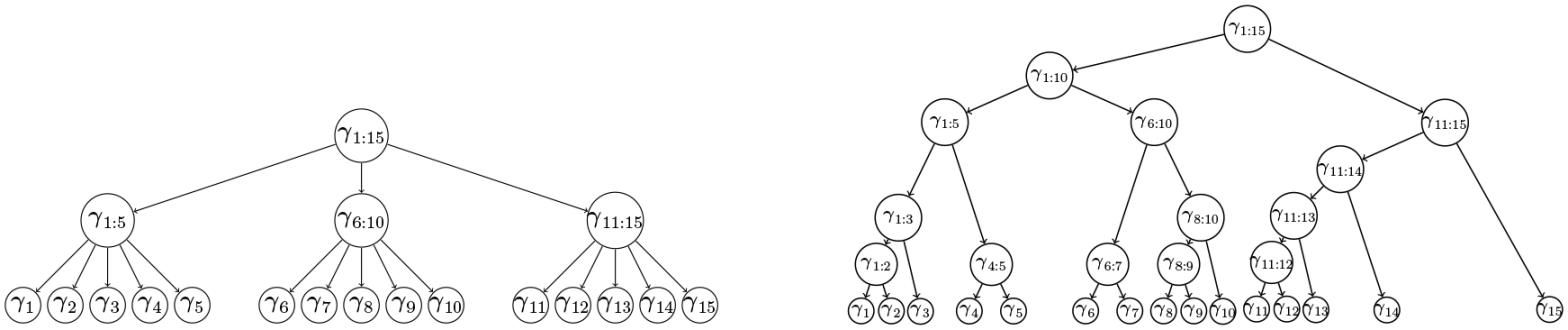
A simple tree used for the “tag-lasso ideal” (left) and a more realistic tree used for the “tag-lasso realistic” (right).

The tag-lasso estimator relies on the existence of a tree to perform node dimension reduction. We consider two different tree structures throughout the simulation study. First, we use an “ideal” tree which contains the true aggregation structure as the sole aggregation level between the leaves and the root of the tree. As an example, the true aggregation structure for the chain graph structure is shown in the left panel of Figure 5. We form A corresponding to this oracle tree to obtain the “*tag-lasso ideal*” estimator.

We also consider a more realistic tree, shown in the right panel of Figure 5, following a construction similar to that of Yan and Bien (2021). The tree is formed by performing hierarchical clustering of p latent points chosen to ensure that the tree contains the true aggregation structure and that these true clusters occur across a variety of depths. In particular, we generate K cluster means $\mu_{1},\ldots ,\mu_{K}$ with $\mu_{i}=1/i$. We set the number of latent points associated with each of the K means equal to the cluster sizes from Figure 4. These latent points are then drawn independently from $N\left(\mu_{i},{\left[0.05\cdot {\text{min}}_{j}\left(\mu_{i}-\mu_{j}\right)\right]}^{2}\right)$. Finally, we form **A** corresponding to this tree to obtain the “*tag-lasso realistic*” estimator.

### 4.2. Results

We subsequently discuss the results on estimation accuracy, aggregation performance, and sparsity recovery.

#### Estimation Accuracy.

**Figure 6:**
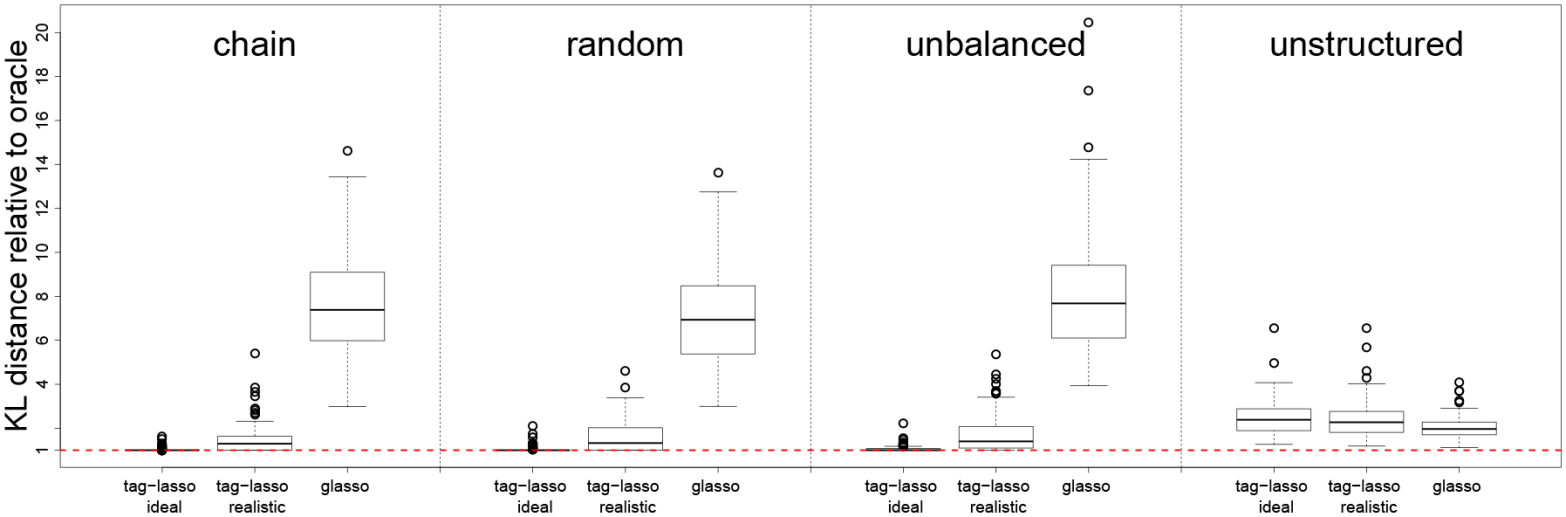
Estimation accuracy of the three estimators relative to the oracle.

Boxplots of the KL distances for the three estimators (tag-lasso ideal, tag-lasso realistic and glasso) relative to the oracle are given in Figure 6. The first three panels correspond to simulation designs with aggregation structures. In these settings, the tag-lasso estimators considerably outperform the glasso, on average by a factor five. The tag-lasso ideal method performs nearly as well as the oracle. Comparing the tag-lasso realistic method to the tag-lasso ideal method suggests a minimal price paid for using a more realistic tree. 

The “unstructured” panel of Figure 6 shows a case in which there is sparsity but no aggregation in the true data generating model. As expected, the glasso performs best in this case: its average KL distance is around 0.41 versus 0.51 for the tag-lasso estimators. We thus observe a minimal cost to applying the tag-lasso, which encompasses the glasso as a special case when $\lambda_{1}=0$. The tag-lasso estimators can indeed reject the additional prior information provided by the tree, thereby selecting a small $\lambda_{1}$ value in the grid, hence a dense $\hat{\Gamma }$ and no node aggregation. In fact, the tag-lasso estimators include on average around 11 nodes in the “aggregated” graph, which is relatively close to the p=15 original variables it should include, thereby explaining the small loss in estimation accuracy compared to the glasso.

**Figure 7:**
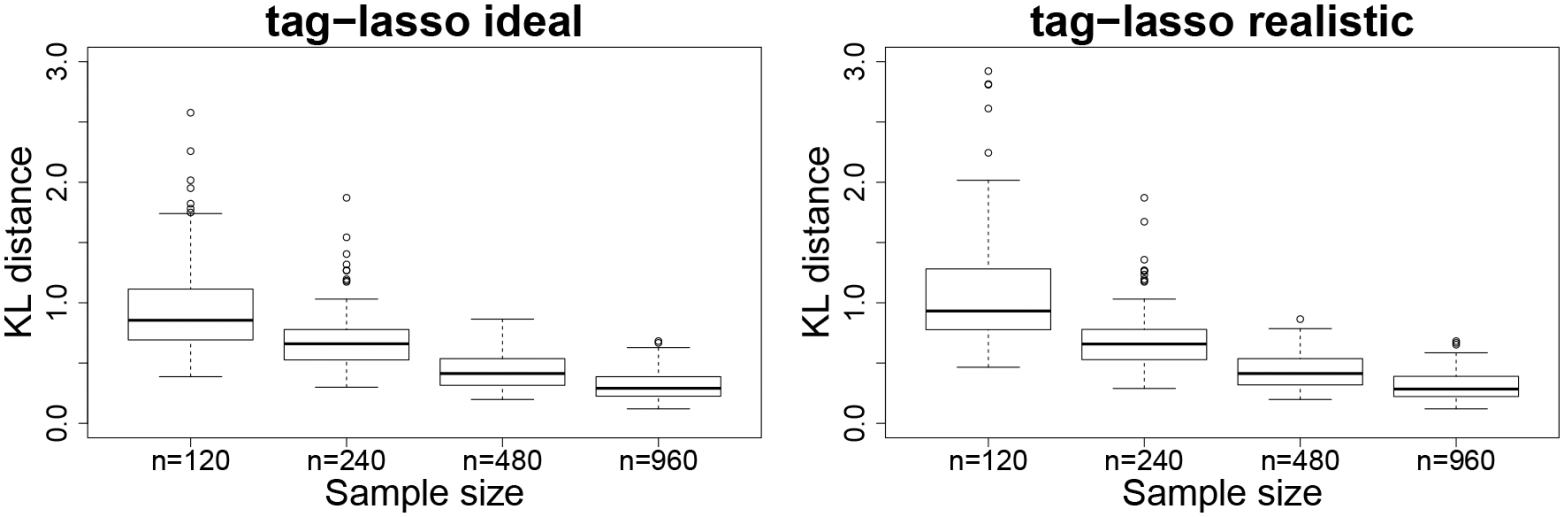
Estimation accuracy of the tag-lasso estimators for the chain design with fixed p=15 and increasing sample size n.

While the paper does not contain theoretical results on the consistency of the tag-lasso estimator, we do investigate this numerically. We consider the chain design with p=15 and increase the sample size from n=120  to  240,480,960. Figure 7 contains the results for both tag-lasso estimators. As expected, we see that the estimation accuracy gradually increases (equivalently the KL distance decreases) for both tag-lasso estimators as the sample size increases relative to the fixed number of variables.

#### Aggregation Performance.

Table 2 summarizes the aggregation performance of the three estimators in terms of the Rand index (RI) and adjusted Rand index (ARI). No results on the ARI in the unstructured simulation design are reported since it cannot be computed for a partition consisting of singletons. The tag-lasso estimators perform very well. If one can rely on an oracle tree, the tag-lasso perfectly recovers the aggregation structure, as reflected in the perfect (A)RI values of the tag-lasso ideal method. Even when the tag-lasso uses a more complex tree structure, it recovers the correct aggregation structure in the vast majority of cases. The glasso returns a partition of singletons as it is unable to perform dimension reduction through aggregation, as can be seen from its zero values on the ARI.

#### Sparsity Recovery.

Table 3 summarizes the results on sparsity recovery (FPR and FNR). The tag-lasso estimators enjoy favorable FPR and FNR, mostly excluding the irrelevant conditional dependencies (as reflected by their low FPR) and including the relevant conditional dependencies (as reflected by their low FNR). In the simulation designs with aggregation, the glasso pays a large price for not being able to reduce dimensionality through aggregation, leading it to include too many irrelevant conditional dependencies, as reflected through its large FPRs. In the unstructured design, the rates of all estimators are, overall, low.

### 4.3. Increasing the Number of Nodes

We investigate the sensitivity of our results to an increasing number of variables p. We focus on the chain simulation design from Section 4.1 and subsequently double p from 15 to 30, 60 and 120 while keeping the number of blocks K fixed at three. The sample size n is set proportional to the complexity of the model, as measured by Kp+p. Hence, the sample sizes corresponding to the increasing values of p are respectively, n=120,240,480,960, thereby keeping the ratio of the sample size to the complexity fixed at two. In each setting, the number of parameters to be estimated is large, equal to 120, 465, 1830, 7260, respectively; thus increasing relative to the sample size.

**Figure 8:**
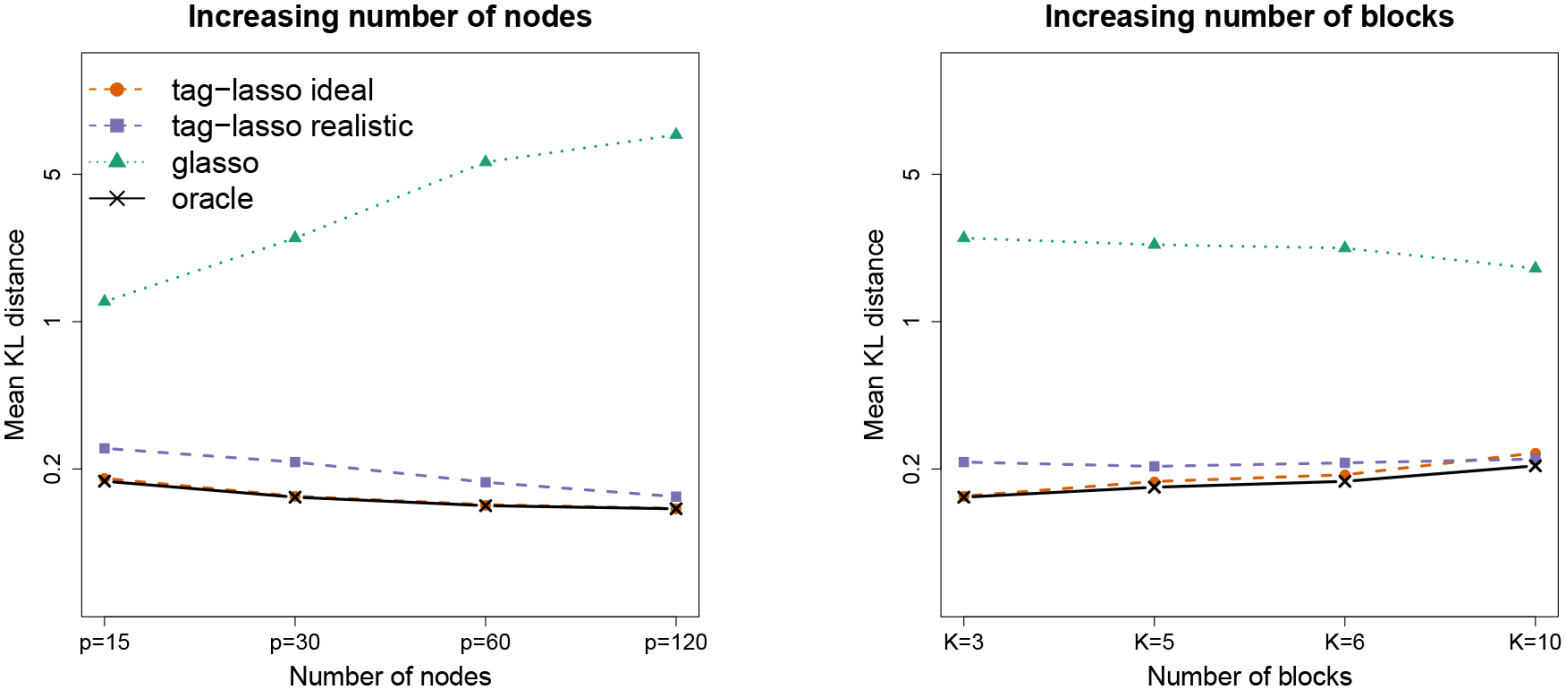
Estimation accuracy of the four estimators (on a log-scale) for increasing number of variables p (and fixed K=3, left panel) the number of blocks K (and fixed p=30, right panel).

The left panel of Figure 8 shows the mean KL distance (on a log-scale) of the four estimators as a function of p. As the number of nodes increases, the estimation accuracy of the tag-lasso estimators and the oracle increases slightly. For fixed K and increasing p, the aggregated nodes—which can be thought of as the average of p/K random variables—may be stabler, thereby explaining why the problem at hand does not get harder when increasing p for the methods with node aggregation. By contrast, the glasso—which is unable to exploit the aggregation structure—performs worse as p increases. For p=120, for instance, the tag-lasso estimators outperform the glasso by a factor 50.

Results on aggregation performance and sparsity recovery are presented in Figure 15 of Appendix D. The tag-lasso ideal method perfectly recovers the aggregation structure for all values of p. The realistic tag-lasso’s aggregation performance is close to perfect and remains relatively stable as p increases. The glasso is unable to detect the aggregation structure, as expected and reflected through its zero ARIs. The tag-lasso estimators also maintain a better balance between the FPR and FNR than the glasso. While their FPRs increase as p increases, their FNRs remain close to perfect, hence all relevant conditional dependencies are recovered. The glasso, in contrast, fails to recover the majority of relevant conditional dependencies when p=60,120, thereby explaining its considerable drop in estimation accuracy.

### 4.4. Increasing the Number of Blocks

Next, we investigate the effect of increasing the number of blocks K. We take the chain simulation design from Section 4.1 and increase the number of blocks from K=3 to K=5,6,10, while keeping the number of variables fixed at p=30. The right panel of Figure 8 shows the mean KL distance (on a log-scale) of the four estimators as a function of K. As one would expect, the difference between the aggregation methods and the glasso decreases as K increases. However, for all K considered, the glasso does far less well than the aggregation based methods.

Similar conclusions hold in terms of aggregation and sparsity recovery performance. Detailed results are presented in Figure 16 of Appendix D. The tag-lasso ideal method performs as well as the oracle in terms of capturing the aggregation structure; the tag-lasso realistic method performs close to perfect and its aggregation performance improves with increasing K. In terms of sparsity recovery, the tag-lasso estimators hardly miss relevant conditional dependencies and only include a small number of irrelevant conditional dependencies. The glasso’s sparsity recovery performance is overall worse but does improve with increasing K.

### 4.5. High-dimensional p>n Design

**Figure 9:**
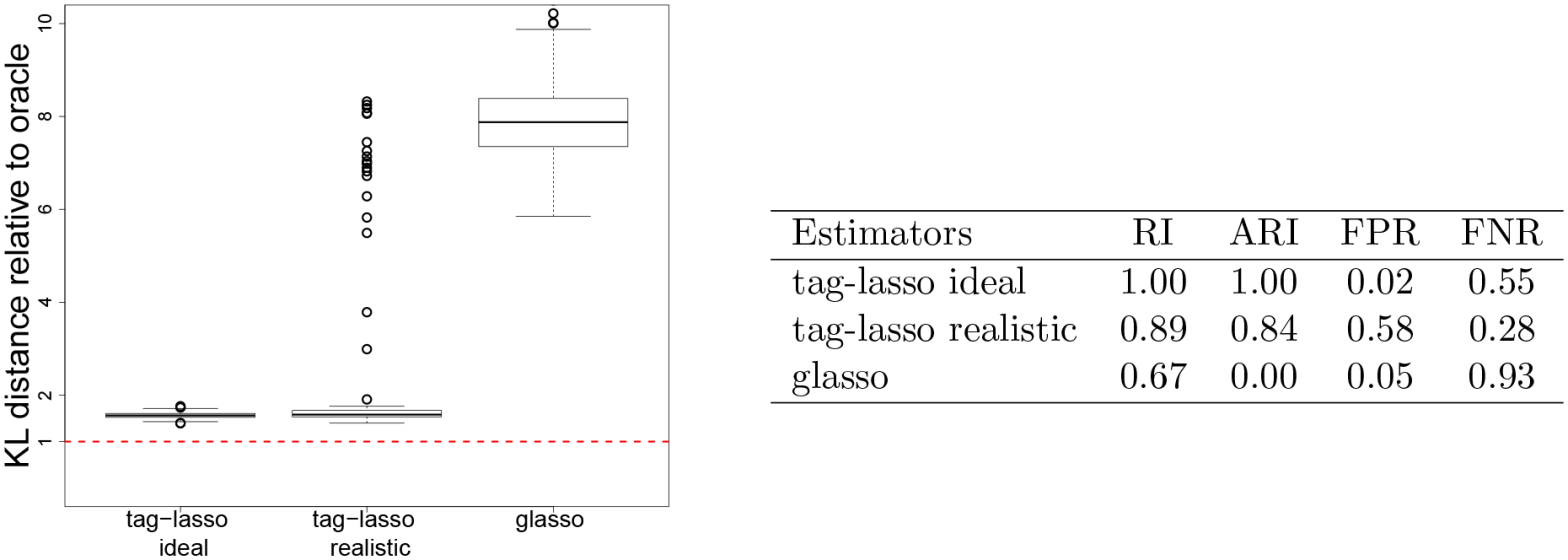
High-dimensional design with p=150,n=120. Left: Estimation accuracy of the three estimators relative to the oracle. Right: Aggregation performance and sparsity recovery of the three estimators. Standard errors around the reported results are all smaller than 0.05, and are thus not reported.

Finally, we investigate the performance of the tag-lasso in a high-dimensional design where the number of variables p exceeds the sample size n. To this end, we consider the chain design with p=150 and n=120. The left panel of Figure 9 presents boxplots of the KL distances of the tag-lasso estimators and glasso relative to the oracle. In this high-dimensional design, the tag-lasso estimators pay a larger price in terms of estimation accuracy compared to the oracle. The same holds for the tag-lasso realistic compared to the tag-lasso ideal. Still both tag-lasso estimators considerably outperform the glasso.

The right panel of Figure 9 summarizes the aggregation performance and sparsity recovery of the tag-lasso estimators and glasso. The aggregation performance of the tag-lasso estimators remains high. Moreover, they balance false positives and false negatives better than the glasso. While the tag-lasso ideal mainly displays a high FNR, the tag-lasso realistic also suffers from returning an overly dense graph, as can be seen from its higher FPR. Glasso, in contrast, cannot handle the many non-zero elements in the true precision matrix in combination with the small sample size. To tackle the dimensionality, it returns an overly sparse solution as can be seen from the high FNR.

## 5. Applications

We consider two applications: a financial (Section 5.1) and a microbiome application (Section 5.2).

### 5.1. Financial Application

**Figure 10:**
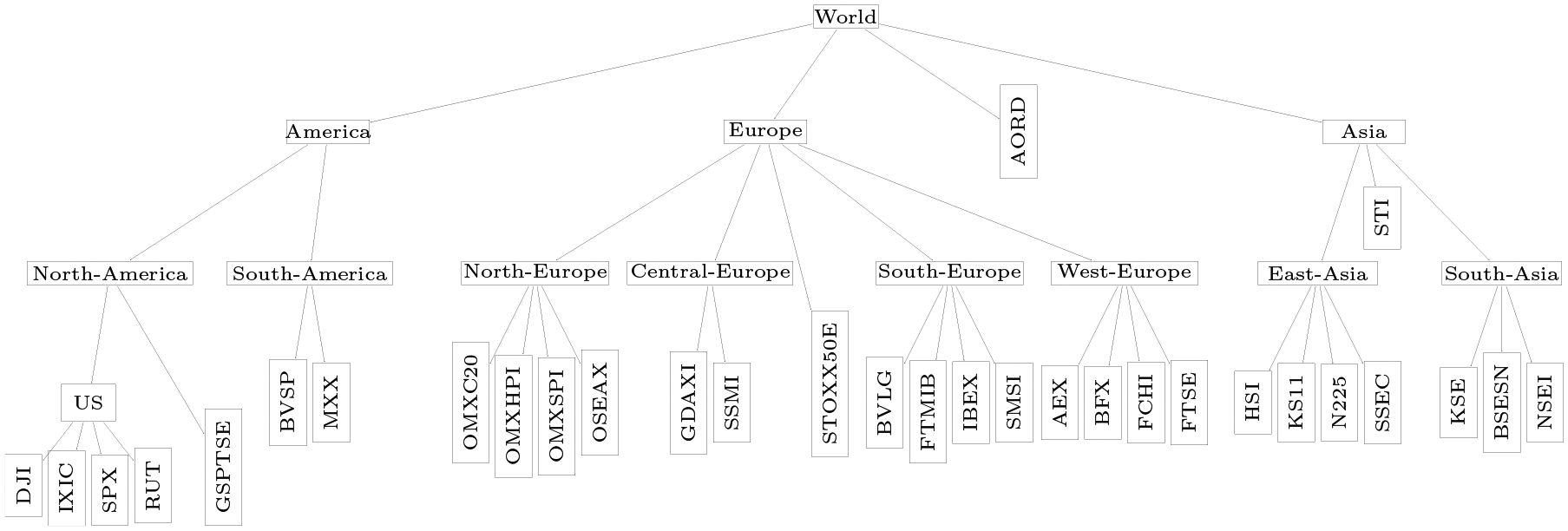
Geography-based tree for the stock market data, which aggregates the p=31 stock market indices (leaves) over several sub-continents towards a single root. Leaves, which represent individual stock markets, are displayed horizontally.

We demonstrate our method on a financial data set containing daily realized variances of p=31 stock market indices from across the world in 2019 (n=254). Daily realized variances based on five minute returns are taken from the Oxford-Man Institute of Quantitative Finance (publicly available at http://realized.oxford-man.ox.ac.uk/data/download). Following standard practice, all realized variances are log-transformed. An overview of the stock market indices is provided in Appendix E. We encode similarity between the 31 stock market indices according to geographical region, and use the tree shown in Figure 10 to apply the tag-lasso estimator.

Since the different observations of the consecutive days are (time)-dependent, we first fit the popular and simple *heterogeneous autoregressive* (HAR) model of (Corsi, 2009) to each of the individual log-transformed realized variance series. Graphical displays of the residual series of these 31 HAR models suggest that almost all autocorrelation in the series is captured. We then apply the tag-lasso to the residual series to learn the conditional dependency structure among stock market indices.

#### Estimated Graphical Model.

**Figure 11:**
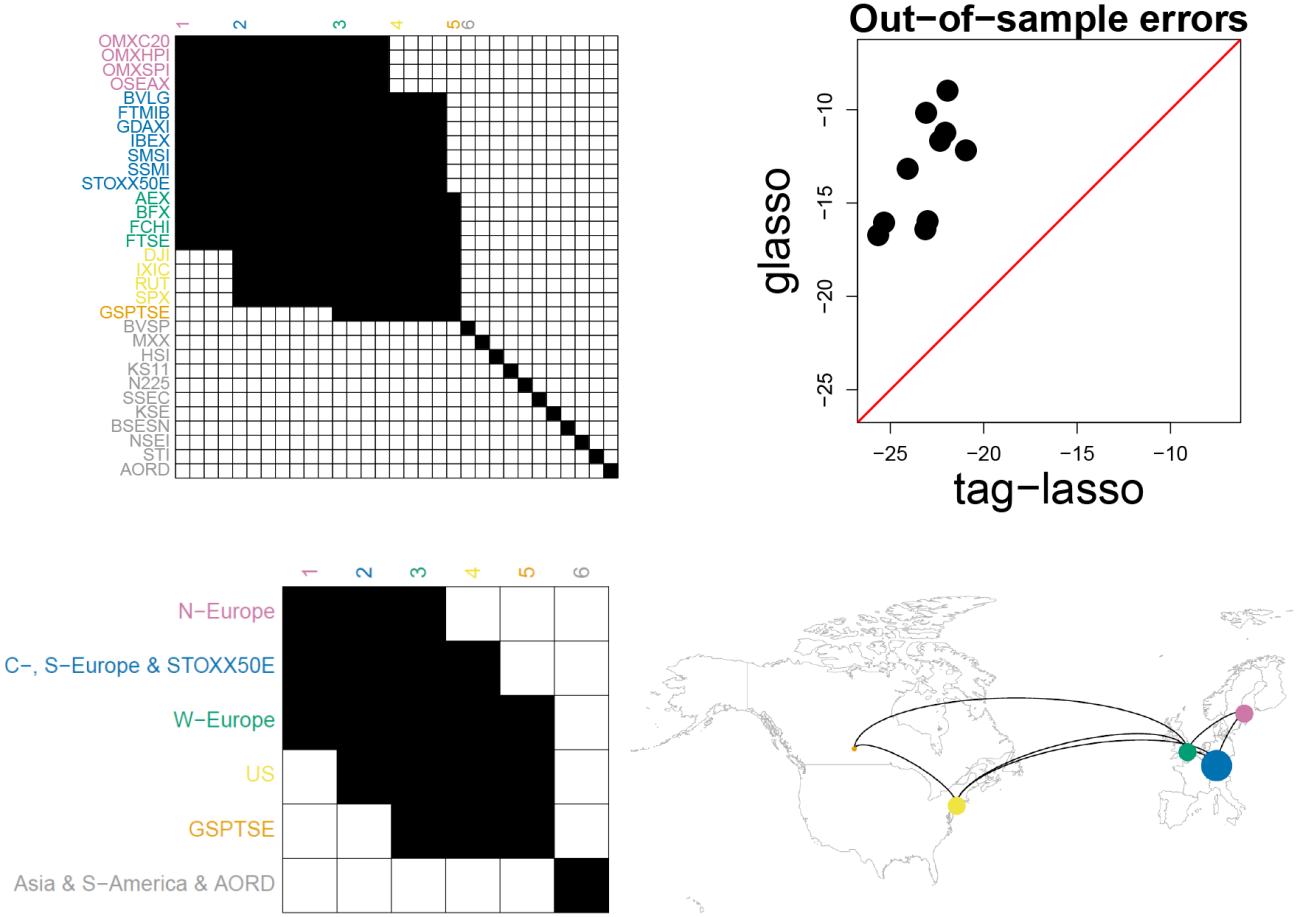
Stock market indices data. Top left: Sparsity pattern (non-zeros in black) of full $\hat{\Omega }$ with aggregation structure conveyed through row label coloring and column numbering. Top right: Test errors across the ten replications (dots) for the tag-lasso versus glasso. Bottom: Aggregated graph for the K=6 nodes obtained with the tag-lasso as an adjacency matrix (bottom left) and as a network (bottom right) with the size of each node proportional to the number of original variables it aggregates.

We fit the tag-lasso estimator, with 5-fold cross-validation to select tuning parameters, to the full data set, with the matrix A encoding the tree structure in Figure 10. The tag-lasso returns a solution with K=6 aggregated blocks; the sparsity pattern of the full estimated precision matrix is shown in the top left panel of Figure 11. The coloring of the row labels and the numbering of columns convey the memberships of each variable to aggregated blocks (to avoid clutter, only the first column of each block is labeled).

Dimension reduction mainly occurs through node aggregation, as can be seen from the aggregated precision matrix in the bottom left panel of Figure 11. The resulting aggregated graphical model is rather dense with only about half of the off-diagonal entries being non-zero in the estimated aggregated precision matrix, thereby suggesting strong volatility connectedness. The solution returned by the tag-lasso estimator consists of one single-market block (block 5: Canada) and five multi-market blocks, which vary in size. The Australian, South-America, and all Asian stock markets form one aggregated block (block 6). Note that the tag-lasso has “aggregated” these merely because they have the same *non*-dependence structure (i.e. all of these markets are estimated to be conditionally inde pendent of each other and all other markets). The remaining aggregated nodes concern the US market (block 4) and three European markets, which are divided into North-Europe (block 1), Central-, South-Europe & STOXX50E (block 2), and West-Europe (block 3). In the aggregated network, the latter two and the US play a central role as they are the most strongly connected nodes: These three nodes are connected to each other, the US node is additionally connected to Canada, whereas these European nodes are additionally connected with North-Europe.

#### Out-of-sample Performance.

We conduct an out-of-sample exercise to compare the tag-lasso estimator to the glasso estimator. We take a random sample of *n* = 203 observations (80% of the full data set) to form a “training sample” covariance matrix and use the remaining data to form a “test sample” covariance matrix $S^{\text{test }}$, and repeat this procedure ten times. We fit both the tag-lasso and glasso estimator to the training covariance matrix, with 5-fold cross-validation on the training data to select tuning parameters. Next, we compute their corresponding out-of-sample errors on the test data, as in (6).

The top right panel of Figure 11 shows each of these ten test errors for both the tag-lasso (x-axis) and the glasso estimator (y-axis). The fact that in all ten replicates the points are well above the 45-degree line indicates that the tag-lasso estimator has better estimation error than the glasso. Tag-lasso has a lower test error than glasso in all ten replicates, resulting in a substantial reduction in glasso’s test errors. This indicates that jointly exploiting edge and node dimension reduction is useful for precision matrix estimation in this context.

### 5.2. Microbiome Application

We next turn to a data set of gut microbial amplicon data in HIV patients (Rivera-Pinto et al., 2018), where our goal is to estimate an interpretable graphical model, capturing the interplay between different taxonomic groups of the microbiome. Bien et al. (2020) recently showed that tree-based aggregation in a supervised setting leads to parsimonious predictive models. The data set has n=152 HIV patients, and we apply the tag-lasso estimator to all p=104 bacterial operational taxonomic units (OTUs) that have non-zero counts in over half of the samples. We use the taxonomic tree that arranges the OTUs into natural hierarchical groupings of taxa: with 17 genera, 11 families, five orders, five classes, three phyla, and one kingdom (the root node). We employ a standard data transformation from the field of compositional data analysis (see e.g., Aitchison, 1982) called the centered log-ratio (clr) transformation that is commonly used in microbiome graphical modeling (Kurtz et al., 2015; Lo and Marculescu, 2018; Kurtz et al., 2019). After transformation, Kurtz et al. (2015) apply the glasso, Lo and Marculescu (2018) incorporate phylogenetic information into glasso’s optimization problem through weights within the $\ell_{1}$-penalty, and Kurtz et al. (2019) estimate a latent graphical model which combines sparsity with a low-rank structure. We instead, use the tag-lasso to learn a sparse aggregated network from the clr-transformed microbiome compositions. While the clr-transform induces dependence between otherwise independent components, Proposition 1 in Cao et al. (2019) provides intuition that as long as the underlying graphical model is sparse and p is large, these induced dependencies may have minimal effect on the covariance matrix. Future work could more carefully account for the induced dependence, incorporating ideas from Cao et al. (2019) or Kurtz et al. (2019).

#### Estimated Graphical Model.

**Figure 12:**
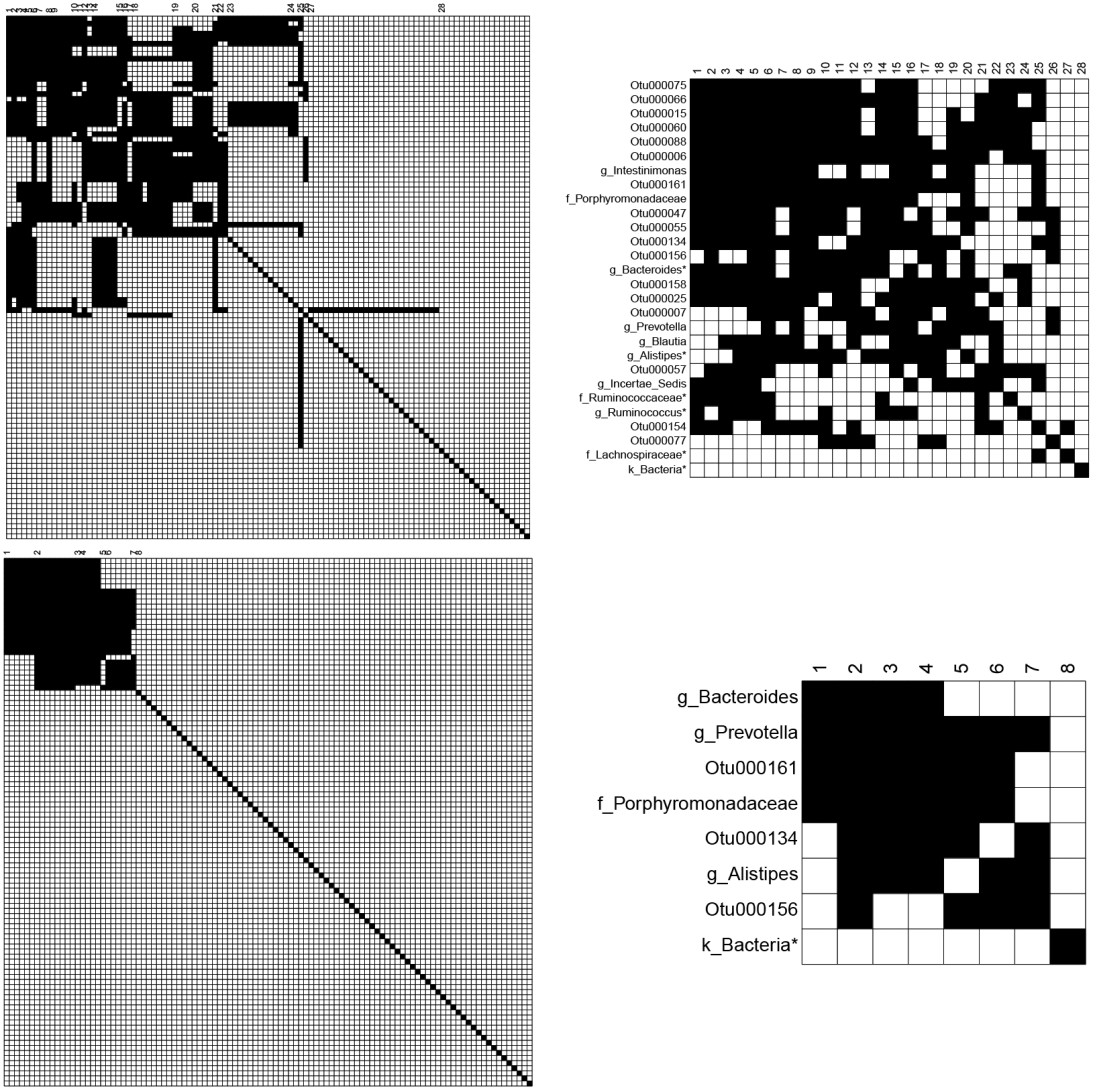
Microbiome data. Full precision matrix (left) and aggregated precision matrix (right) estimated by the tag-lasso with an unconstrained five-fold cross-validation (top) and with a cross-validation subject to the constraint that there are at most ten blocks (bottom).

We fit the tag-lasso to the full data set and use 5-fold cross-validation to select the tuning parameters. The tag-lasso estimator provides a sparse aggregated graphical model with K=28 aggregated blocks (a substantial reduction in nodes from the original p=104 OTUs). The top panel of Figure 12 shows the sparsity pattern of the p×p estimated precision matrix (top left) and of the K×K estimated aggregated precision matrix (top right). A notable feature of the tag-lasso solution is that it returns a wide range of aggregation levels: The aggregated network consists of 17 OTUs, 7 nodes aggregated to the genus level (these nodes start with “g_”), 3 to the family level (these nodes start with “f_”), and 1 node to the kingdom level (this node starts with “k_”). Some aggregated nodes, such as the “g_Blautia” node (block 19), contain all OTUs within their taxa; some other aggregated nodes, indicated with an asterisk like the “k_Bacteria*” node (block 28), have some of their OTUs missing. This latter “block” consists of 18 OTUs from across the phylogenetic tree that are estimated to be conditionally independent with all other OTUs in the data set.

While the tag-lasso determines the aggregation level in a data-driven way through crossvalidation, practitioners or researchers may also sometimes wish to restrict the number of blocks K to a pre-determined level when such prior knowledge is available or if this is desirable for interpretability. As an illustration, we consider a *constrained cross-validation* scheme in which we restrict the number of blocks K to maximally ten and select the sparsity parameters with the best cross-validated error among those solutions with K≤10. The bottom panel of Figure 12 shows the sparsity pattern of the full and aggregated precision matrices estimated by this constrained version of the tag-lasso.

**Figure 13:**
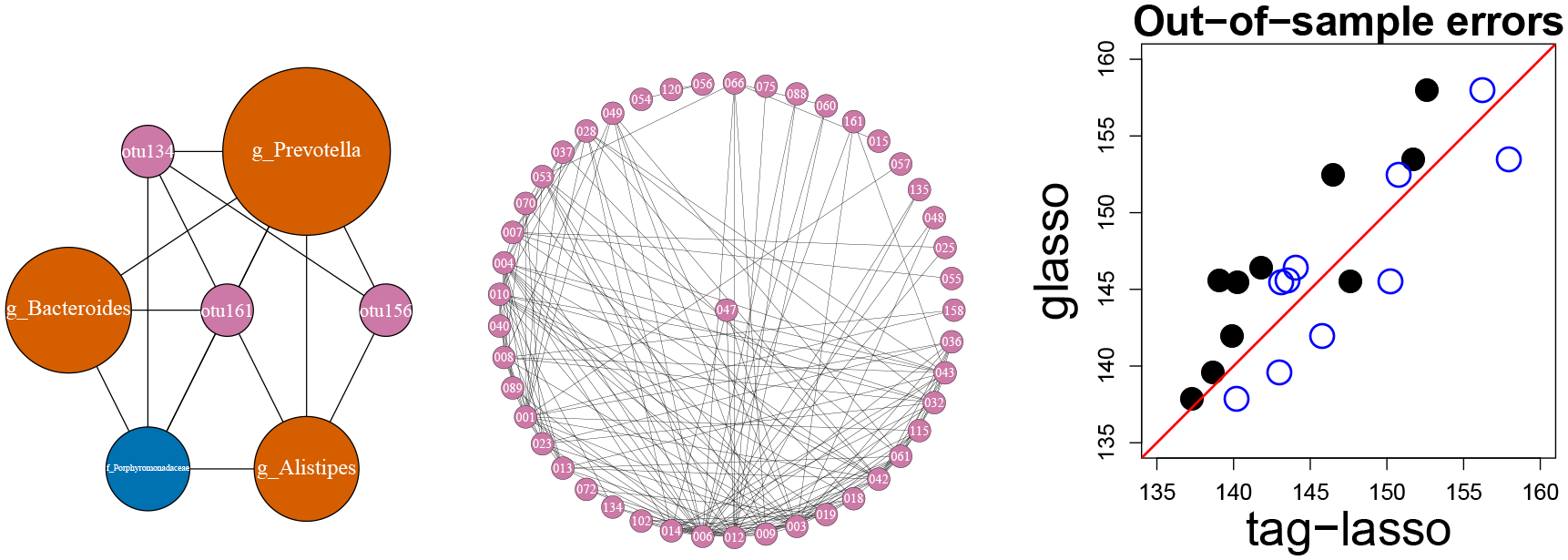
Microbiome data. Left: Aggregated network estimated by the constrained CV version of the tag-lasso. The colour of the nodes is based on their level of aggregation (OTU: pink, genus: orange, family: blue); their width is proportional to the number of OTUs they aggregate. Middle: Network estimated by the glasso. Right: Test errors across the ten replications for the unconstrained (solid black) and constrained (unfilled blue) CV version of the tag-lasso versus the glasso.

**Figure 15:**
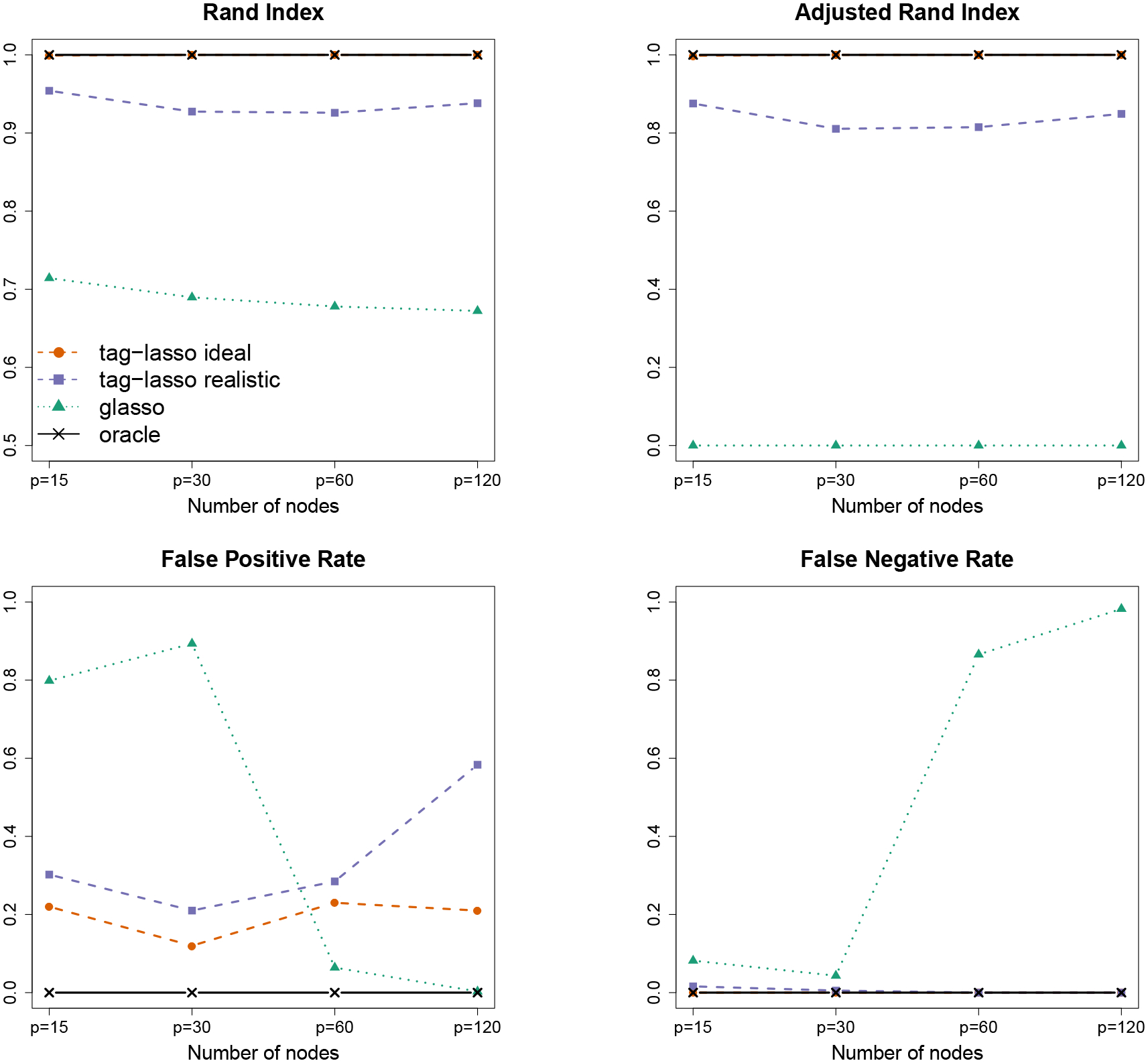
Simulation results for increasing number of nodes p. Top: Aggregation performance (RI: left; ARI: right); Bottom: Sparsity recovery (FPR: left; FNR: right) of the four estimators

**Figure 16:**
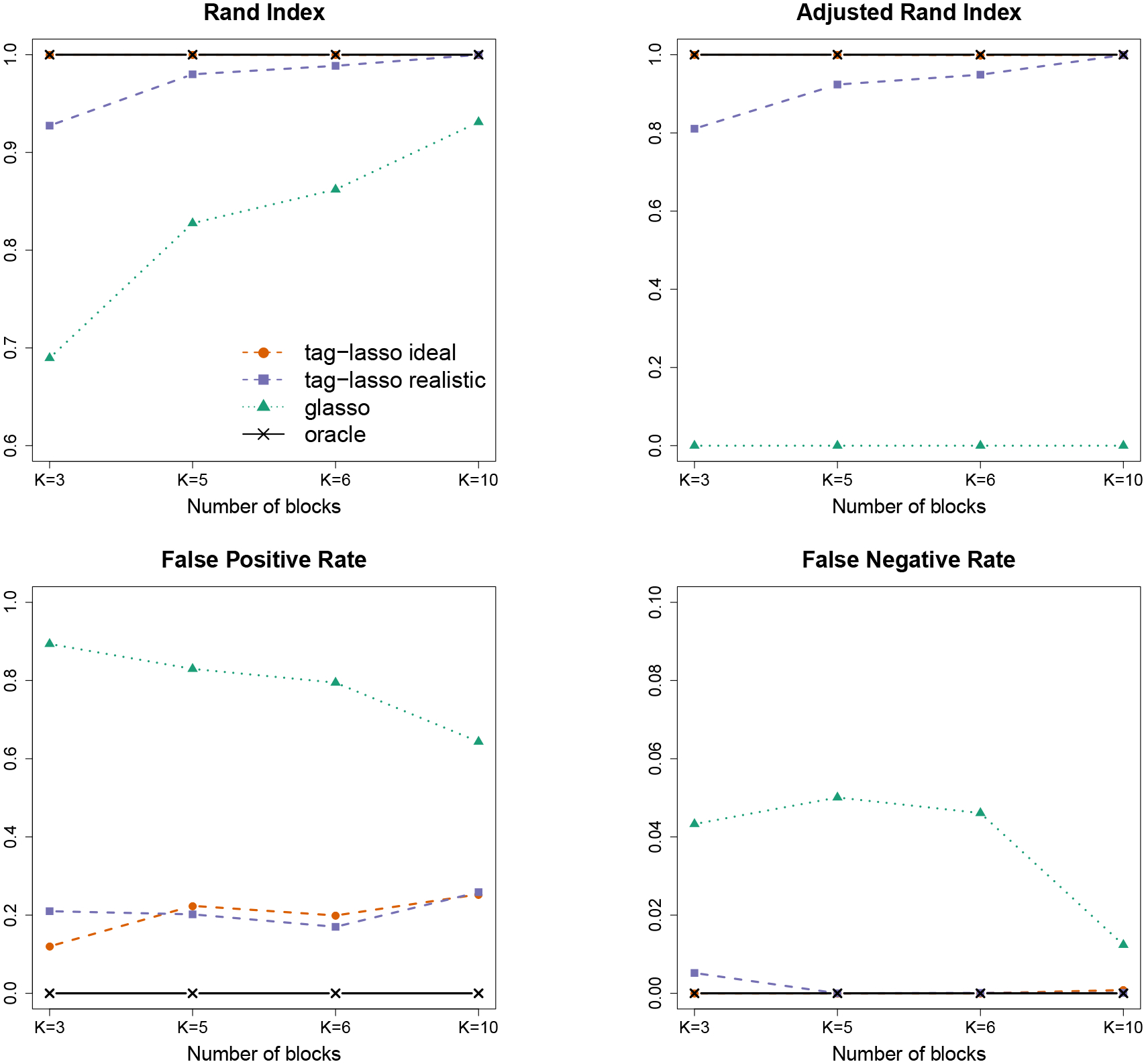
Simulation results for increasing number of blocks K. Top: Aggregation performance (RI: left; ARI: right); Bottom: Sparsity recovery (FPR: left; FNR: right) of the four estimators

The resulting network consists of K=8 aggregated nodes. The “k_Bacteria*” node now aggregates 78 OTUs that are estimated to be conditionally independent of each other and all others. The interactions among the remaining nodes are shown in the left panel of Figure 13, which consists of three OTUs (OTU134, OTU156, and OTU161, in pink), three genera (Prevotella, Bacteroides, and Alistipes in orange) and one family (Porphyromonadaceae in blue). The resulting network is much simpler than the one estimated by the glasso, shown in the middle panel of Figure 13. The glasso finds 58 OTUs to be conditionally independent with all others, but the interactions among the remaining 46 OTUs are much more difficult to interpret. The glasso is limited to working at the OTU-level, which prevents it from providing insights about interactions that span different levels of the taxonomy.

#### Out-of-sample Performance.

We conduct the same out-of-sample exercise as described in Section 5.1. The right panel of Figure 13 presents the ten test errors (black dots) for the unconstrained CV tag-lasso and glasso. In all but one case, the tag-lasso leads to a better fit than the glasso, suggesting that it is better suited for modeling the conditional dependencies among the OTUs. The unfilled blue dots show the same but for the constrained CV tag-lasso. In all ten cases, it underperforms the unconstrained CV tag-lasso (see shift to the right on the horizontal axis); however, its performance is on a par with the glasso, with test errors close to the 45 degree line. Thus, there does not appear to be a cost in out-of-sample-performance to the interpretability gains of the constrained tag-lasso over the glasso.

## 6. Conclusion

Detecting conditional dependencies between variables, as represented in a graphical model, forms a cornerstone of multivariate data analysis. However, graphical models, characterized by a set of nodes and edges, can quickly explode in dimensionality due to ever-increasing fine-grained levels of resolution at which data are measured. In many applications, a tree is available that organizes the measured variables into various meaningful levels of resolution. In this work, we introduce the tag-lasso, a novel estimation procedure for graphical models that curbs this curse of dimensionality through joint node and edge dimension reduction by leveraging this tree as side information. Node dimension reduction is achieved by a penalty that allows nodes to be aggregated according to the tree structure; edge dimension reduction is achieved through a standard sparsity-inducing penalty. As such, the tag-lasso generalizes the popular glasso approach to sparse graphical modelling. An R package called taglasso implements the proposed method and is available on the GitHub page (https://github.com/ineswilms/taglasso) of the first author.

The tree is a crucial ingredient for performing node aggregation with the tag-lasso and opens up several interesting avenues for future research. On the one hand, multiple trees could be available. For instance, we could think of aggregating stock data by sector, transaction volume, or capitalization. In such cases, it would be interesting to let the trees compete in, for instance, a cross-validation exercise from which the tree that “best” fits the data can be selected to guide the node aggregation. On the other hand, some applications lack the availability of a tree but have more general graph-based structures available that could guide the node aggregation. To this end, it would be interesting to further investigate how the machinery of graph fusion penalties (see e.g., Wang et al., 2016) could be leveraged.

Finally, we do not provide theory in the form of estimation error bounds for the tag-lasso estimator. Possible starting directions to this end can be found in Rothman et al. (2008) or Ravikumar et al. (2011). It would be interesting to investigate the effect of different tree structures on the bounds. In Ravikumar et al. (2011), for instance, the maximal node degree is a relevant quantity. For our penalty on Ω, which involves an internal optimization problem over Γ and D, it would be interesting to understand the relevant quantity that captures the interplay between the tree structure and the true node aggregation structure.

## Figures and Tables

**Figure 14:**
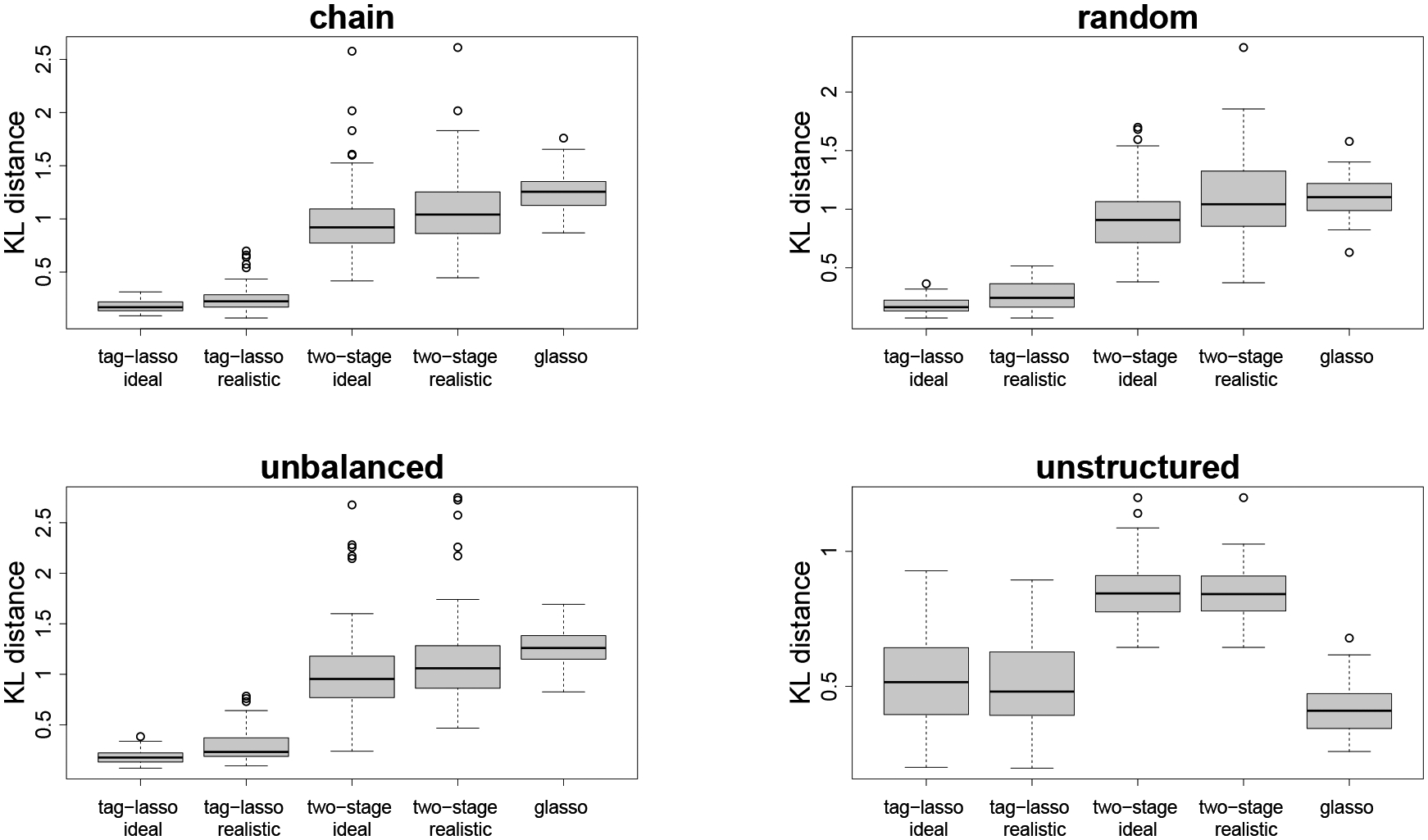
Estimation accuracy of the tag-lasso estimators compared to the two-stage estimators and glasso.

**Table 1:** Toy example: Covariance and precision matrices with corresponding graphical model (drawn for p=50) for the full (top) and aggregated (bottom) set of nodes.

Nodes	Covariance Matrix Σ	Precision Matrix Ω	Graphical Model
$X_{1},\ldots ,X_{p}$	$\left(\begin{array}{ccc} p-1 & p-2 & 1_{p-2}^{⊤}\\ p-2 & p-1 & 1_{p-2}^{⊤}\\ 1_{p-2} & 1_{p-2} & I_{p-2} \end{array}\right)$	$\left(\begin{array}{ccc} 1 & 0 & -1_{p-2}^{⊤}\\ 0 & 1 & -1_{p-2}^{⊤}\\ -1_{p-2} & -1_{p-2} & L \end{array}\right)$ with $L=I_{p-2}+2\cdot 1_{p-2}\cdot 1_{p-2}^{⊤}$	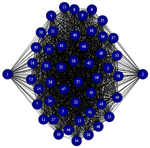
$X_{1},X_{2},\tilde{X}$	$\left(\begin{array}{ccc} p-1 & p-2 & p-2\\ p-2 & p-1 & p-2\\ p-2 & p-2 & p-2 \end{array}\right)$	$\left(\begin{array}{ccc} 1 & 0 & -1\\ 0 & 1 & -1\\ -1 & -1 & 2+1/(p-2) \end{array}\right)$	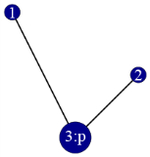

Note: Let $1_{d}$ denote a d-dimensional column vector of ones, and $I_{d}$ be the d × d identity matrix.

**Table 2:** Aggregation performance of the three estimators, as measured by the Rand index (RI) and adjusted Rand index (ARI), for the four simulation designs. Standard errors are in parentheses.

Estimators	chain		random		unbalanced		unstructured	
	RI	ARI	RI	ARI	RI	ARI	RI	ARI
tag-lasso ideal	1.00 (.00)	1.00 (.01)	1.00 (.00)	1.00 (.00)	1.00 (.00)	0.99 (.01)	0.84 (.02)	NA
tag-lasso realistic	0.95 (.01)	0.88 (.01)	0.97 (.01)	0.93 (.01)	0.94 (.01)	0.85 (.02)	0.81 (.02)	NA
glasso	0.71 (.00)	0.00 (.00)	0.71 (.00)	0.00 (.00)	0.67 (.00)	0.00 (.00)	1.00 (.00)	NA

**Table 3:** Sparsity recovery of the three estimators, as measured by the false positive rate (FPR) and false negative rate (FNR), for the four simulation designs. Standard errors are in parentheses

Estimators	chain		random		unbalanced		unstructured	
	FPR	FNR	FPR	FNR	FPR	FNR	FPR	FNR
tag-lasso ideal	0.22 (.04)	0.00 (.00)	0.19 (.04)	0.00 (.01)	0.46 (.05)	0.00 (.00)	0.06 (.01)	0.15 (.01)
tag-lasso realistic	0.30 (.04)	0.02 (.01)	0.13 (.02)	0.09 (.01)	0.44 (.04)	0.05 (.01)	0.05 (.01)	0.14 (.01)
glasso	0.80 (.02)	0.08 (.01)	0.73 (.01)	0.09 (.01)	0.82 (.02)	0.07 (.01)	0.16 (.01)	0.04 (.01)

## Appendix A. Proof of Propositions

### A.1 Proof of Proposition 1

**Proof** First, note that $\tilde{X}$ follows a K-dimensional multivariate normal distribution with mean zero and covariance matrix $M^{⊤}{\left(D+MCM^{⊤}\right)}^{-1}M$. Next, we re-write this covariance matrix by two successive applications of equation (23) in Henderson and Searle (1981):

$$M^{⊤}{\left(D+MCM^{⊤}\right)}^{-1}M=M^{⊤}D^{-1}M-M^{⊤}D^{-1}M{\left(I+CM^{⊤}D^{-1}M\right)}^{-1}CM^{⊤}D^{-1}M\;={\left({\left[M^{⊤}D^{-1}M\right]}^{-1}+C\right)}^{-1}.$$

Hence, the precision matrix of $\tilde{X}$ is given by ${\left(M^{⊤}D^{-1}M\right)}^{-1}+C$. Now since ${\left(M^{⊤}D^{-1}M\right)}^{-1}$ is diagonal, $c_{ij}=0\Leftrightarrow {\tilde{X}}_{i}⊥{\tilde{X}}_{j}|{\tilde{X}}_{-\left\{i,j\right\}}$, for any i,j=1,…,K and with ${\tilde{X}}_{-\left\{i,j\right\}}$ containing all aggregated variables except for aggregate i and j. ∎

### A.2 Proof of Proposition 2

**Proof** Denote the tag-lasso solution by $\left(\hat{\Omega },\hat{\Gamma },\hat{D}\right)$ and let $\hat{𝒱}=\left\{u:{\hat{\gamma }}_{u}\neq 0\right\}$ be the set of non-zero rows in $\hat{\Gamma }$. Define the partial ordering over the nodes of the tree so that u≤v means that u∉ancestor(v) and label the nodes of $\hat{𝒱}$ so that $u_{1}\leq u_{2}\leq \cdots \leq u_{\left|\hat{𝒱}\right|}$.

Consider the following algorithm to obtain the partition matrix $\hat{M}$. We work our way through the nodes in $\hat{𝒱}$ in a bottom-up fashion (according to the tree). In a Gram-Schmidtlike procedure, for each node, we subtract out the contributions of its descendants. This approach achieves orthogonality while preserving the binary matrix structure. Note that the approach may result in one or more zero columns in the partition matrix, which are removed at the end before returning the final partition matrix $\hat{M}$.

To prove that $\hat{M}$ is a partition matrix, we need to show that it is a binary matrix with ${\hat{M}}^{⊤}\hat{M}$ a diagonal matrix (i.e., orthogonal columns). Let ${𝒯}_{u}$ denote the subtree rooted at u∈𝒯. For any $u\in \hat{𝒱}$, equation (8) implies that

(9)
$$A_{u}=M_{u}+\underset{u^{\prime }\in \text{descendant}\left(u\right)\cap \hat{𝒱}}{\sum }M_{u^{\prime }}=\underset{u^{\prime }\in {𝒯}_{u}\cap \hat{𝒱}}{\sum }M_{u^{\prime }}.$$

By equation (9), we can then re-write equation (8) as

(10)
$$M_{a}=A_{a}-\underset{u\in \text{children}\left(a\right)}{\sum }\underset{u^{\prime }\in {𝒯}_{u}\cap \hat{𝒱}}{\sum }M_{u^{\prime }}\;=A_{a}-\underset{u\in \text{children}\left(a\right)}{\sum }A_{u}.$$

$\text{Now supp}\left(A_{u}\right)\cap \text{supp}\left(A_{v}\right)=\emptyset$ for u,v∈children(a) and u≠v, and with supp() denoting the support. Using that $\text{supp}\left(A_{u}\right)\subseteq \text{supp}\left(A_{a}\right)$ for u∈children(a), it follows that $M_{a}\in {\left\{0,1\right\}}^{p}$ and $\text{supp}\left(M_{a}\right)\subseteq \text{supp}\left(A_{a}\right)$.

Now, consider $a,b\in \hat{𝒱}$, a≠b with the following three cases:

1. If b∈descendant(a), then
  (11)
  $$M_{b}^{⊤}M_{a}=M_{b}^{⊤}\left(A_{a}-\underset{u^{\prime }\in \text{descendant}\left(a\right)\cap \hat{𝒱}}{\sum }M_{u^{\prime }}\right)\;\;=M_{b}^{⊤}A_{b}-M_{b}^{⊤}\underset{u^{\prime }\in \text{descendant}\left(a\right)\cap \hat{𝒱}}{\sum }M_{u^{\prime }}\;=M_{b}^{⊤}A_{b}^{⊤}-M_{b}^{⊤}\underset{u^{\prime }\in {𝒯}_{b}\cap \hat{𝒱}}{\sum }M_{u}^{\text{'}},\;=M_{b}^{⊤}A_{b}^{⊤}-M_{b}^{⊤}A_{b}^{⊤}\;=0.$$
  The second line follows from $\text{supp}\left(M_{b}\right)\subseteq \text{supp}\left(A_{b}\right)\subseteq \text{supp}\left(A_{a}\right)$; the third line follows since $\text{supp}\left(M_{b}\right)\subseteq \text{supp}\left(A_{b}\right)$ and descendant $\left(a\right)\cap {𝒯}_{b}={𝒯}_{b}$ thereby recalling that b∈descendant(a); and the fourth line follows from equation (9).
2. If a∈descendant(b), then $M_{b}^{⊤}M_{a}=0$ follows from case 1.
3. If a and b are in disjoint branches, then $\text{supp}\left(A_{a}\right)\cap \text{supp}\left(A_{b}\right)=\emptyset$ so $M_{a}^{⊤}M_{b}=0$.

We have thus constructed a partition matrix $\hat{M}$, namely $\hat{M}\in {\left\{0,1\right\}}^{p\times K}$ with ${\hat{M}}^{⊤}\hat{M}$ diagonal. By construction ${\hat{M}}_{u}$ is in the column space of $A_{\hat{𝒱}}$, denoted as ${\hat{M}}_{u}\in {ℒ}_{\text{col }}\left(A_{\hat{𝒱}}\right)$, for each $u\in \hat{𝒱}$. Hence, ${ℒ}_{\text{col }}\left(\hat{M}\right)\subseteq {ℒ}_{\text{col }}\left(A_{\hat{𝒱}}\right)$ It then follows that

$$\hat{\Omega }-\hat{D}=A\hat{\Gamma }=A_{\hat{𝒱}}{\hat{\Gamma }}_{\hat{𝒱}}\in {ℒ}_{\text{col}}(\hat{M}).$$

By symmetry of $\hat{\Omega }-\hat{D},\hat{\Omega }-\hat{D}$ is also in the row space of $\hat{M}$. We can therefore write

$$\hat{\Omega }-\hat{D}=\hat{M}{\hat{M}}^{+}\left(\hat{\Omega }-\hat{D}\right){\left({\hat{M}}^{+}\right)}^{⊤}{\hat{M}}^{⊤},$$

where ${\hat{M}}^{+}={\left({\hat{M}}^{⊤}\hat{M}\right)}^{-1}{\hat{M}}^{⊤}$ is the projection matrix onto the column space of $\hat{M}$. Taking $\hat{C}={\hat{M}}^{+}\left(\hat{\Omega }-\hat{D}\right){\left({\hat{M}}^{+}\right)}^{⊤}$ then establishes the result. ∎

## Appendix B. Tag-lasso Estimator Compared to a Two-stage Benchmark

We compare the performance of the tag-lasso estimator to a two-stage benchmark. For the benchmark, we first apply the tag-lasso estimator with $\lambda_{2}=0$ to solely determine the level of node aggregation. Secondly, we apply the graphical lasso with a hard aggregation constraint (similar to the one used in equation (7)), as provided by the outcome of the first stage. We use the simulation designs detailed in Section 4.1 to compare the estimation accuracy of this two-stage estimator to the tag-lasso and regular glasso. For the tag-lasso and two-stage estimators both the ideal and realistic tree structure are used.

Figure 14 presents the results on KL distance for the five estimators across the four simulation designs. The tag-lasso estimators provide, overall, a considerable improvement in terms of estimation accuracy over their two-stage benchmarks. For the simulation designs where node aggregation is present (chain, random, unbalanced), the two-stage estimators do still outperform the glasso on average.

## Appendix C. Details of the LA-ADMM Algorithm

The augmented Lagrangian of (5) is given by

(12)
$$-\text{logdet}\left(\Omega^{\left(1\right)}\right)+\text{tr}\left(S\Omega^{\left(1\right)}\right)+1_{\infty }\left\{\Omega^{\left(1\right)}=\Omega^{\left(1\right)}{\;}^{⊤},\Omega^{\left(1\right)}≻0\right\}+\langle U^{\left(1\right)},\Omega^{\left(1\right)}-\Omega \rangle +\frac{\rho }{2}{\left\|\Omega^{\left(1\right)}-\Omega \right\|}_{F}^{2}+\lambda_{1}{\left\|\Gamma_{-r}^{\left(1\right)}\right\|}_{2,1}+1_{\infty }\left\{\gamma_{r}^{\left(1\right)}=\gamma^{\left(1\right)}1_{p}\right\}+\langle U^{\left(4\right)},\Gamma^{\left(1\right)}-\Gamma \rangle +\frac{\rho }{2}{\left\|\Gamma^{\left(1\right)}-\Gamma \right\|}_{F}^{2}+1_{\infty }\left\{\Omega^{\left(2\right)}=A\Gamma^{\left(2\right)}+D,D\text{ diag}.,D_{jj}\geq 0\right\}+\langle U^{\left(2\right)},\Omega^{\left(2\right)}-\Omega \rangle +\frac{\rho }{2}{\left\|\Omega^{\left(2\right)}-\Omega \right\|}_{F}^{2}+\langle U^{\left(5\right)},\Gamma^{\left(2\right)}-\Gamma \rangle +\frac{\rho }{2}{\left\|\Gamma^{\left(2\right)}-\Gamma \right\|}_{F}^{2}+\lambda_{2}{\left\|\Omega^{-\text{diag}\left(3\right)}\right\|}_{1}+\langle U^{\left(3\right)},\Omega^{\left(3\right)}-\Omega \rangle +\frac{\rho }{2}{\left\|\Omega^{\left(2\right)}-\Omega \right\|}_{F}^{2},$$

where $U^{\left(i\right)}$ (for i=1,…,5) are the dual variables, and ρ is a penalty parameter. Note that equation (12) is of the same form as Equation (3.1) in Boyd et al. (2011) and thus involves iterating three basic steps: (i) minimization with respect to $\left(\Omega^{\left(1\right)},\Omega^{\left(2\right)},\Omega^{\left(3\right)},\Gamma^{\left(1\right)},\Gamma^{\left(2\right)},D\right)$, (ii) minimization with respect to (Ω,Γ), and (iii) update of $\left(U^{\left(1\right)},\ldots ,U^{\left(5\right)}\right)$.

Step (i) decouples into four independent problems, whose solutions are worked out in Sections C.1–C.4. Step (ii) involves the minimization of a differentiable function of Ω and Γ and boils down to the calculation of simple averages, as shown in Section C.5. Step (iii)’s update of the dual variables is provided in C.6

Algorithms 2–3 then provide an overview of the LA-ADMM algorithm to solve problem (5). We use the LA-ADMM algorithm with $\rho_{1}=0.01,{\text{ T}}_{\text{stages }}=10$, maxit=100.

### C.1 Solving for $\Omega^{\left(1\right)}$

Minimizing the augmented Lagrangian with respect to $\Omega^{\left(1\right)}$ gives ${\hat{\Omega }}_{k+1}^{\left(1\right)}$, the solution to the optimization problem

$$\underset{\Omega^{\left(1\right)}}{\text{min}}\left\{-\text{logdet}\left(\Omega^{\left(1\right)}\right)+\text{tr}\left(S\Omega^{\left(1\right)}\right)+U^{\left(1\right)},\Omega^{\left(1\right)}-\Omega +\frac{\rho }{2}{\left\|\Omega^{\left(1\right)}-\Omega \right\|}_{F}^{2}\text{ s}.\text{t}.\;\Omega^{\left(1\right)}=\Omega^{{\left(1\right)}^{⊤}},\Omega^{\left(1\right)}≻0\right\}=\underset{\Omega^{\left(1\right)}}{\text{min}}\left\{-\text{logdet}\left(\Omega^{\left(1\right)}\right)+\text{tr}\left(S\Omega^{\left(1\right)}\right)+\frac{\rho }{2}{\left\|\Omega^{\left(1\right)}-\left({\hat{\Omega }}_{k}-{\hat{U}}_{k}^{\left(1\right)}/\rho \right)\right\|}_{F}^{2}\text{ s}.\text{t}.\;\Omega^{\left(1\right)}=\Omega^{\left(1\right)},\Omega^{\left(1\right)}≻0\right\}.$$

The solution should satisfy the first order optimality condition

(13)
$$\rho {\hat{\Omega }}_{k+1}^{\left(1\right)}-{\left({\hat{\Omega }}_{k+1}^{\left(1\right)}\right)}^{-1}=\rho {\hat{\Omega }}_{k}-{\hat{U}}_{k}^{\left(1\right)}-S.$$

This means that the eigenvectors of ${\hat{\Omega }}_{k+1}^{\left(1\right)}$ are the same as the eigenvectors of $\rho {\hat{\Omega }}_{k}-{\hat{U}}_{k}^{\left(1\right)}-S$ and that the eigenvalues of ${\hat{\Omega }}_{k+1}^{\left(1\right)}$ are a simple function of the eigenvalues of $\rho {\hat{\Omega }}_{k}-{\hat{U}}_{k}^{\left(1\right)}-S$. Consider the orthogonal eigenvalue decomposition of right hand side:

$$\rho {\hat{\Omega }}_{k}-{\hat{U}}_{k}^{\left(1\right)}-S=Q\Lambda Q^{⊤},$$

where $\Lambda =\text{diag}\left(\delta_{1},\ldots ,\delta_{p}\right)$ and $QQ^{⊤}=Q^{⊤}Q=I$. Multiply (13) by $Q^{⊤}$ on the left and Q on the right

$$\rho {\bar{\Omega }}_{k+1}^{\left(1\right)}-{\left({\bar{\Omega }}_{k+1}^{\left(1\right)}\right)}^{-1}=\Lambda ,\text{ with }{\bar{\Omega }}_{k+1}^{\left(1\right)}=Q^{⊤}{\hat{\Omega }}_{k+1}^{\left(1\right)}Q.$$

Then

(14)
$${\hat{\Omega }}_{k+1}^{\left(1\right)}=Q{\bar{\Omega }}_{k+1}^{\left(1\right)}Q^{⊤},\text{ with }{\bar{\Omega }}_{k+1,jj}^{\left(1\right)}=\frac{\delta_{j}+\sqrt{\delta_{j}^{2}+4\rho }}{2\rho }.$$

### C.2 Solving for $\Gamma^{\left(1\right)}$

Minimizing the augmented Lagrangian with respect to $\Gamma^{\left(1\right)}$ gives

$${\hat{\Gamma }}_{k+1}^{\left(1\right)}≔\underset{\Gamma^{\left(1\right)}}{\text{argmin}}\left\{\frac{\rho }{2}{\left\|\Gamma^{\left(1\right)}-\left({\hat{\Gamma }}_{k}-{\hat{U}}_{k}^{\left(4\right)}/\rho \right)\right\|}_{F}^{2}+\lambda_{1}{\left(\Gamma_{-r}^{\left(1\right)}\right]}_{2,1}\text{ s}.\text{t}.\;\gamma_{r}^{\left(1\right)}=\gamma^{\left(1\right)}1_{p}\right\}.$$

The solution is groupwise soft-thresholding:

(15)
$${\hat{\Gamma }}_{k+1,j}^{\left(1\right)}=\{\begin{array}{ll} S_{G}\left({\hat{\Gamma }}_{k,j}-{\hat{U}}_{k,j}^{\left(4\right)}/\rho ,\lambda_{1}/\rho \right), & \text{ if }j=1,\ldots ,\left|𝒯\right|\setminus \left\{r\right\}\\ {\hat{\gamma }}_{k}1_{p}, & \text{ if }j=r. \end{array}$$

with the group-wise soft-thresholding operator $S_{G}\left(\gamma ,\lambda \right)=\text{max}\left(1-\lambda /∥\gamma {∥}_{2},0\right)\gamma$ applied to $\gamma \in {\mathbb{R}}^{p}$, and ${\hat{\gamma }}_{k}$ is equal to the average of the p-dimensional vector ${\hat{\Gamma }}_{k,r}-{\hat{U}}_{k,r}^{\left(4\right)}/\rho$. Note that in this Appendix we use the capitalized $\Gamma_{j}$ notation to index the $j^{\text{th }}$ row of the matrix Γ whereas we use lowercase $\gamma_{u}$ when indexing a node u based on the tree structure in Section 2 of the main paper.

### C.3 Solving for $\Omega^{\left(2\right)},\Gamma^{\left(2\right)},D$

Minimizing the augmented Lagrangian with respect to $\Omega^{\left(2\right)},\Gamma^{\left(2\right)},D$ gives

$$\left({\hat{\Omega }}_{k+1}^{\left(2\right)},{\hat{\Gamma }}_{k+1}^{\left(2\right)},{\hat{D}}_{k+1}\right)≔\underset{\Omega^{\left(2\right)},\Gamma^{\left(2\right)},D}{\text{argmin}}\left\{\frac{\rho }{2}{\left\|\Omega^{\left(2\right)}-\left({\hat{\Omega }}_{k}-{\hat{U}}_{k}^{\left(2\right)}/\rho \right)\right\|}_{F}^{2}+\frac{\rho }{2}{\left\|\Gamma^{\left(2\right)}-\left({\hat{\Gamma }}_{k}-{\hat{U}}_{k}^{\left(5\right)}/\rho \right)\right\|}_{F}^{2}\text{s}.\text{t}.\;\Omega^{\left(2\right)}=A\Gamma^{\left(2\right)}+D,D\text{ diagonal, }D_{jj}\geq 0\text{ for }j=1,\ldots ,p.\right\}$$

The solution

(16)
$${\hat{\Omega }}_{k+1}^{\left(2\right)}=A{\hat{\Gamma }}_{k+1}^{\left(2\right)}+{\hat{D}}_{k+1}$$

is immediate and we are left with

$$\left({\hat{\Gamma }}_{k+1}^{\left(2\right)},{\hat{D}}_{k+1}\right)≔\underset{\Gamma^{\left(2\right)},D}{\text{argmin}}\left\{\frac{1}{2}{\left\|\tilde{A}\Gamma^{\left(2\right)}+\tilde{D}-\tilde{M}\right\|}_{F}^{2}\text{ s}.\text{t}.\;D\text{ diagonal, }D_{jj}\geq 0\text{ for }j=1,\ldots ,p\right\}$$

where we have substituted $\Omega^{\left(2\right)}=A\Gamma^{\left(2\right)}+D$ and we denote

$$\tilde{A}=\left(\begin{array}{c} A\\ I_{\left|𝒯\right|} \end{array}\right)\in {\mathbb{R}}^{\left(p+\left|𝒯\right|\right)\times \left|𝒯\right|},\tilde{D}=\left(\begin{array}{c} D\\ 0_{\left|𝒯\right|\times p} \end{array}\right)\in {\mathbb{R}}^{\left(p+\left|𝒯\right|\right)\times p},\text{ and }\tilde{M}=\left(\begin{array}{c} {\hat{\Omega }}_{k}-{\hat{U}}_{k}^{\left(2\right)}/\rho \\ {\hat{\Gamma }}_{k}-{\hat{U}}_{k}^{\left(5\right)}/\rho \end{array}\right)\in {\mathbb{R}}^{\left(p+\left|𝒯\right|\right)\times p}.$$

The solution

(17)
$${\hat{\Gamma }}_{k+1}^{\left(2\right)}={\left({\tilde{A}}^{⊤}\tilde{A}\right)}^{-1}{\tilde{A}}^{⊤}\left(\tilde{M}-{\tilde{D}}_{k+1}\right)\;={\left(A^{⊤}A+I_{\left|𝒯\right|}\right)}^{-1}\left(A^{⊤}:I_{\left|𝒯\right|}\right)\left(\tilde{M}-{\tilde{D}}_{k+1}\right)$$

is immediate and we are left with

$${\hat{D}}_{k+1}\;≔\underset{D}{\text{argmin}}\left\{\frac{1}{2}{\left\|\left(\tilde{M}-\tilde{D}\right)-\tilde{A}{\left({\tilde{A}}^{⊤}\tilde{A}\right)}^{-1}{\tilde{A}}^{⊤}\left(\tilde{M}-\tilde{D}\right)\right\|}_{F}^{2}\text{ s}.\text{t}.\;D\text{ diag}.,\;D_{jj}\geq 0,j=1,\ldots ,p,\right\}\;=\underset{D}{\text{argmin}}\left\{\frac{1}{2}{\left\|\left(I_{p+\left|𝒯\right|}-\tilde{A}{\left({\tilde{A}}^{⊤}\tilde{A}\right)}^{-1}{\tilde{A}}^{⊤}\right)\left(\tilde{M}-\tilde{D}\right)\right\|}_{F}^{2}\text{ s}.\text{t}.\;D\text{ diag}.,\;D_{jj}\geq 0,j=1,\ldots ,p,\right\}\;=\underset{D}{\text{argmin}}\left\{\frac{1}{2}∥B-CD{∥}_{F}^{2}\text{ s}.\text{t}.\;D\text{ diag}.,\;D_{jj}\geq 0,j=1,\ldots ,p,\right\},$$

with $B=\left(I_{p+\left|𝒯\right|}-\tilde{A}{\left({\tilde{A}}^{⊤}\tilde{A}\right)}^{-1}{\tilde{A}}^{⊤}\right)\tilde{M}\in {\mathbb{R}}^{\left(p+\left|𝒯\right|\right)\times p}$, $C={\left(I_{p}:0_{p\times \left|𝒯\right|}\right)}^{⊤}-\tilde{A}{\left({\tilde{A}}^{⊤}\tilde{A}\right)}^{-1}A^{⊤}\in {\mathbb{R}}^{\left(p+\left|𝒯\right|\right)\times p}$. The solution is

(18)
$$\text{diag}\left({\hat{D}}_{k+1}\right)=\text{diag}{\left(C^{⊤}C\right)}^{-1}\text{diag}{\left(B^{⊤}C\right)}_{+}\cdot$$

### C.4 Solving for $\Omega^{\left(3\right)}$

Minimizing the augmented Lagrangian with respect to $\Omega^{\left(3\right)}$ gives

$${\hat{\Omega }}_{k+1}^{\left(3\right)}\;≔\underset{\Omega^{\left(3\right)}}{\text{argmin}}\left\{\langle U^{\left(3\right)},\Omega^{\left(3\right)}-\Omega \rangle +\frac{\rho }{2}{\left\|\Omega^{\left(3\right)}-\Omega \right\|}_{F}^{2}+\lambda_{2}{\left\|\Omega^{-\text{diag}\left(3\right)}\right\|}_{1}\right\}\;=\underset{\Omega^{\left(3\right)}}{\text{argmin}}\left\{\frac{\rho }{2}{\left\|\Omega^{\left(3\right)}-\left({\hat{\Omega }}_{k}-{\hat{U}}_{k}^{\left(3\right)}/\rho \right)\right\|}_{F}^{2}+\frac{1}{2\rho }{\left\|U^{\left(3\right)}\right\|}_{F}^{2}+\lambda_{2}{\left\|\Omega^{-\text{diag}\left(3\right)}\right\|}_{1}\right\}\;=\underset{\Omega^{\left(3\right)}}{\text{argmin}}\left\{\frac{\rho }{2}{\left\|\Omega^{\left(3\right)}-\left({\hat{\Omega }}_{k}-{\hat{U}}_{k}^{\left(3\right)}/\rho \right)\right\|}_{F}^{2}+\lambda_{2}{\left\|\Omega^{-\text{diag}\left(3\right)}\right\|}_{1}\right\}.$$

The solution is simply elementwise soft-thresholding:

(19)
$${\hat{\Omega }}_{k+1,ij}^{\left(3\right)}=\{\begin{array}{ll} S\left({\hat{\Omega }}_{k,ij}-{\hat{U}}_{k,ij}^{\left(3\right)}/\rho ,\lambda_{2}/\rho \right), & \text{ if }i\neq j\\ {\hat{\Omega }}_{k,ij}-{\hat{U}}_{k,ij}^{\left(3\right)}/\rho , & \text{ if }i=j, \end{array}$$

with the soft-threshold operator S(ω,λ)=sign(ω)max(|ω|−λ,0) applied to ω∈ℝ.

### C.5 Update Variables Ω and Γ

Minimizing the augmented Lagrangian with respect to variables Ω and Γ gives

(20)
$${\hat{\Omega }}_{k+1}≔\underset{\Omega }{\text{argmin}}\left\{\overset{3}{\underset{i=1}{\sum }}{\left\|{\hat{\Omega }}_{k+1}^{\left(i\right)}-\left(\Omega -{\hat{U}}_{k}^{\left(i\right)}/\rho \right)\right\|}_{F}^{2}\right\}={\bar{\Omega }}_{k+1}+\frac{1}{\rho }{\bar{U}}_{k}^{\Omega }$$

(21)
$${\hat{\Gamma }}_{k+1}≔\underset{\Gamma }{\text{argmin}}\left\{\overset{2}{\underset{i=1}{\sum }}{\left\|{\hat{\Gamma }}_{k+1}^{\left(i\right)}-\left(\Gamma -{\hat{U}}_{k}^{\left(i+3\right)}/\rho \right)\right\|}_{F}^{2}\right\}={\bar{\Gamma }}_{k+1}+\frac{1}{\rho }{\bar{U}}_{k}^{\Gamma },$$

where ${\bar{\Omega }}_{k}≔\frac{{\hat{\Omega }}_{k}^{\left(1\right)}+{\hat{\Omega }}_{k}^{\left(2\right)}+{\hat{\Omega }}_{k}^{\left(3\right)}}{3},{\bar{U}}_{k}^{\Omega }≔\frac{{\hat{U}}_{k}^{\left(1\right)}+{\hat{U}}_{k}^{\left(2\right)}+{\hat{U}}_{k}^{\left(3\right)}}{3},{\bar{\Gamma }}_{k}≔\frac{{\hat{\Gamma }}_{k}^{\left(1\right)}+{\hat{\Gamma }}_{k}^{\left(2\right)}}{2},{\bar{U}}_{k}^{\Gamma }≔\frac{{\hat{U}}_{k}^{\left(4\right)}+{\hat{U}}_{k}^{\left(5\right)}}{2}$.

### C.6 Update Dual Variables

The updates of the dual variables are given by

$${\hat{U}}_{k+1}^{\left(i\right)}≔{\hat{U}}_{k}^{\left(i\right)}+\rho \left({\hat{\Omega }}_{k+1}^{\left(i\right)}-{\hat{\Omega }}_{k+1}\right),\text{ for }i=1,\ldots ,3$$

$${\hat{U}}_{k+1}^{\left(j+3\right)}≔{\hat{U}}_{k}^{\left(j+3\right)}+\rho \left({\hat{\Gamma }}_{k+1}^{\left(j\right)}-{\hat{\Gamma }}_{k+1}\right),\text{ for }j=1,\ldots ,2.$$

Similarly, averaging the first three updates and the latter two gives

(22)
$${\bar{U}}_{k+1}^{\Omega }≔{\bar{U}}_{k}^{\Omega }+\rho \left({\bar{\Omega }}_{k+1}-{\hat{\Omega }}_{k+1}\right),\text{ for }i=1,\ldots ,3$$

(23)
$${\bar{U}}_{k+1}^{\Gamma }≔{\bar{U}}_{k}^{\Gamma }+\rho \left({\bar{\Gamma }}_{k+1}-{\hat{\Gamma }}_{k+1}\right),\text{ for }j=1,\ldots ,2.$$

Substituting (20) and (21) into (22) and (23) yields that ${\bar{U}}_{k+1}^{\Omega }={\bar{U}}_{k+1}^{\Gamma }=0$ after the first iteration.

## Appendix E. Financial Application: Data Description

**Table 4:** Financial Application: Data Description, as taken from https://realized.oxford-man.ox.ac.uk/data/assets

Abbreviation	Description	Location
DJI	Dow Jones Industrial Average	US
IXIC	Nasdaq 100	US
SPX	S&P 500 Index	US
RUT	Russel 2000	US
GSPTSE	S&P/TSX Composite index	Canada
BVSP	BVSP BOVESPA Index	Brazil
MXX	IPC Mexico	Mexico
OMXC20	OMX Copenhagen 20 Index	Denmark
OMXHPI	OMX Helsinki All Share Index	Finland
OMXSPI	OMX Stockholm All Share Index	Sweden
OSEAX	Oslo Exchange All-share Index	Norway
GDAXI	Deutscher Aktienindex	Germany
SSMI	Swiss Stock Market Index	Switzerland
BVLG	Portuguese Stock Index	Portugal
FTMIB	Financial Times Stock Exchange Milano Indice di Borsa	Italy
IBEX	Iberia Index 35	Spain
SMSI	General Madrid Index	Spain
AEX	Amsterdam Exchange Index	Netherlands
BFX	Bell 20 Index	Belgium
FCHI	Cotation Assistee en Continue 40	France
FTSE	Financial Times Stock Exchange 100	UK
STOXX50E	EURO STOXX 50	Europe
HSI	HANG SENG Index	Hong Kong
KS11	Korea Composite Stock Price Index (KOSPI)	South Korea
N225	Nikkei 225	Japan
SSEC	Shanghai Composite Index	China
STI	Straits Times Index	Singapore
KSE	Karachi SE 100 Index	Pakistan
BSESN	S&P Bombay Stock Exchange Sensitive Index	India
NSEI	NIFTY 50	India
AORD	All Ordinaries Index	Australia
